# Transient astrocytic mGluR5 expression drives synaptic plasticity and subsequent chronic pain in mice

**DOI:** 10.1084/jem.20210989

**Published:** 2022-03-23

**Authors:** Yosuke Danjo, Eiji Shigetomi, Yukiho J. Hirayama, Kenji Kobayashi, Tatsuya Ishikawa, Yugo Fukazawa, Keisuke Shibata, Kenta Takanashi, Bijay Parajuli, Youichi Shinozaki, Sun Kwang Kim, Junichi Nabekura, Schuichi Koizumi

**Affiliations:** 1 Department of Neuropharmacology, Interdisciplinary Graduate School of Medicine, University of Yamanashi, Yamanashi, Japan; 2 Yamanashi GLIA Center, University of Yamanashi, Yamanashi, Japan; 3 Department of Functional Anatomy, Graduate School of Medical Science, Kanazawa University, Kanazawa, Japan; 4 Division of Brain Structure and Function, Faculty of Medical Sciences, University of Fukui, Fukui, Japan; 5 Department of Physiology, College of Korean Medicine, Kyung Hee University, Seoul, Korea; 6 Division of Homeostatic Development, National Institute for Physiological Sciences, Okazaki, Aichi, Japan; 7 Department of Physiological Sciences, The Graduate School for Advanced Study, Hayama, Kanagawa, Japan

## Abstract

Activation of astrocytes has a profound effect on brain plasticity and is critical for the pathophysiology of several neurological disorders including neuropathic pain. Here, we show that metabotropic glutamate receptor 5 (mGluR5), which reemerges in astrocytes in a restricted time frame, is essential for these functions. Although mGluR5 is absent in healthy adult astrocytes, it transiently reemerges in astrocytes of the somatosensory cortex (S1). During a limited spatiotemporal time frame, astrocytic mGluR5 drives Ca^2+^ signals; upregulates multiple synaptogenic molecules such as Thrombospondin-1, Glypican-4, and Hevin; causes excess excitatory synaptogenesis; and produces persistent alteration of S1 neuronal activity, leading to mechanical allodynia. All of these events were abolished by the astrocyte-specific deletion of mGluR5. Astrocytes dynamically control synaptic plasticity by turning on and off a single molecule, mGluR5, which defines subsequent persistent brain functions, especially under pathological conditions.

## Teaser

In the early, limited time frame of neuropathic pain, mGluR5 reemerges in cortical astrocytes, causing drastic increases in synapse and network remodeling, leading to intractable mechanical allodynia. Despite its restricted expression, astrocytic mGluR5 plays a determinant role in subsequent brain functions.

## Graphical Abstract

**Figure:**
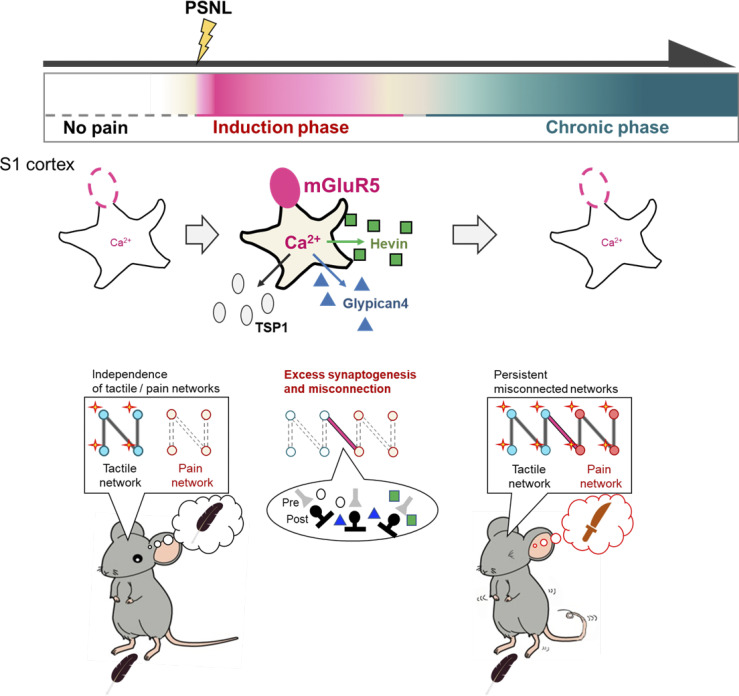

## Introduction

Neuropathic pain, a chronic and refractory type of pain arising from peripheral or central nerve injury, is often associated with mechanical allodynia, whereby a normal “soft” touch is felt as noxious pain (Eisenstein, 2016). Neuropathic pain is a serious disease that affects approximately one fifth of the global population, but its treatment is difficult, and nonsteroidal anti-inflammatory drugs or opioids can have limited beneficial effects. Although the pathogenic mechanisms of neuropathic pain have not been clarified, recent findings clearly show that glial cells, especially microglia and astrocytes in the spinal cord, undergo marked functional changes that are associated with neuropathic pain (Beggs et al., 2012; Shibata et al., 2011; Tsuda et al., 2011; Tsuda et al., 2003). In addition, supraspinal regions, including the primary somatosensory (S1) cortex, have been recognized as critical for pain perception, because significant changes in their function and morphology were observed in humans and animals with abnormal pain perception (Bushnell et al., 1999; Hu et al., 2015).

We recently reported that in a mouse model of neuropathic pain, astrocytes in the S1 cortex played an essential role in the induction of mechanical allodynia (Kim et al., 2016; Kim et al., 2017). Partial sciatic nerve ligation (PSNL) in mice caused mechanical allodynia, which consisted of two phases: an induction phase, in which the pain gradually increased 1 wk after PSNL, followed by a chronic phase, in which the maximum pain persisted for at least several weeks. After PSNL, astrocytes in the S1 cortex become reactive and transform into a synaptogenic phenotype. S1 astrocytes showed increased Ca^2+^ activity during the induction phase, produced thrombospondin-1 (TSP1), and caused excess synapse formation and misconnection between tactile and pain networks, leading to mechanical allodynia. Increased Ca^2+^ excitation initiates this series of responses, because the pharmacological inhibition or genetic deletion of type 2 IP_3_ receptors ameliorated TSP1 production in astrocytes, synaptogenesis, and mechanical allodynia (Kim et al., 2016; Kim et al., 2017).

Although metabotropic glutamate receptor 5 (mGluR5) is suggested to be involved in PSNL-induced Ca^2+^ fluctuation and TSP1 production in S1 astrocytes, it remains unclear how and whether mGluR5 actually plays an essential role in the above events, because mGluR5 is reported to be almost absent in mature astrocytes (Cahoy et al., 2008; Rusnakova et al., 2013; Sun et al., 2013). mGluR5 expression has been reported to reemerge in adult astrocytes in rodents and human patients during some pathological conditions including brain ischemia (Buscemi et al., 2017), Alzheimer’s disease (Grolla et al., 2013; Iyer et al., 2014; Shrivastava et al., 2013), Down syndrome (Iyer et al., 2014), amyotrophic lateral sclerosis (Anneser et al., 2004; Rossi et al., 2008), and epilepsy (Umpierre et al., 2016). Therefore, we hypothesized that mGluR5 may reemerge in S1 astrocytes after PSNL during the induction phase of pain, leading to excess synapse formation, network remodeling, and mechanical allodynia. At present, it remains unknown whether, when, and how mGluR5 reappearance in S1 cortical astrocytes causes neuropathic pain. To answer these questions, we generated brain astrocyte–specific mGluR5 conditional knockout (astro-mGluR5-cKO) mice.

In this study, we show the crucial role of astrocytic mGluR5 in the synaptic reorganization involved in induction of neuropathic pain. After PSNL, mGluR5 reemerged in S1 astrocytes within a critical temporal window for pathogenesis of neuropathic pain, the so-called induction phase. We demonstrate that mGluR5 reemergence in S1 astrocytes was responsible for induction of synaptogenic molecules, excess synapse formation, and mechanical allodynia. We report that astrocytes could dramatically enhance synapse plasticity even in the adult brain, which was achieved by the spatial and temporal restriction of the reemergence of a single molecule, mGluR5, in astrocytes.

## Results

### Transient reemergence of mGluR5 in S1 astrocytes after PSNL

**Figure 1.**
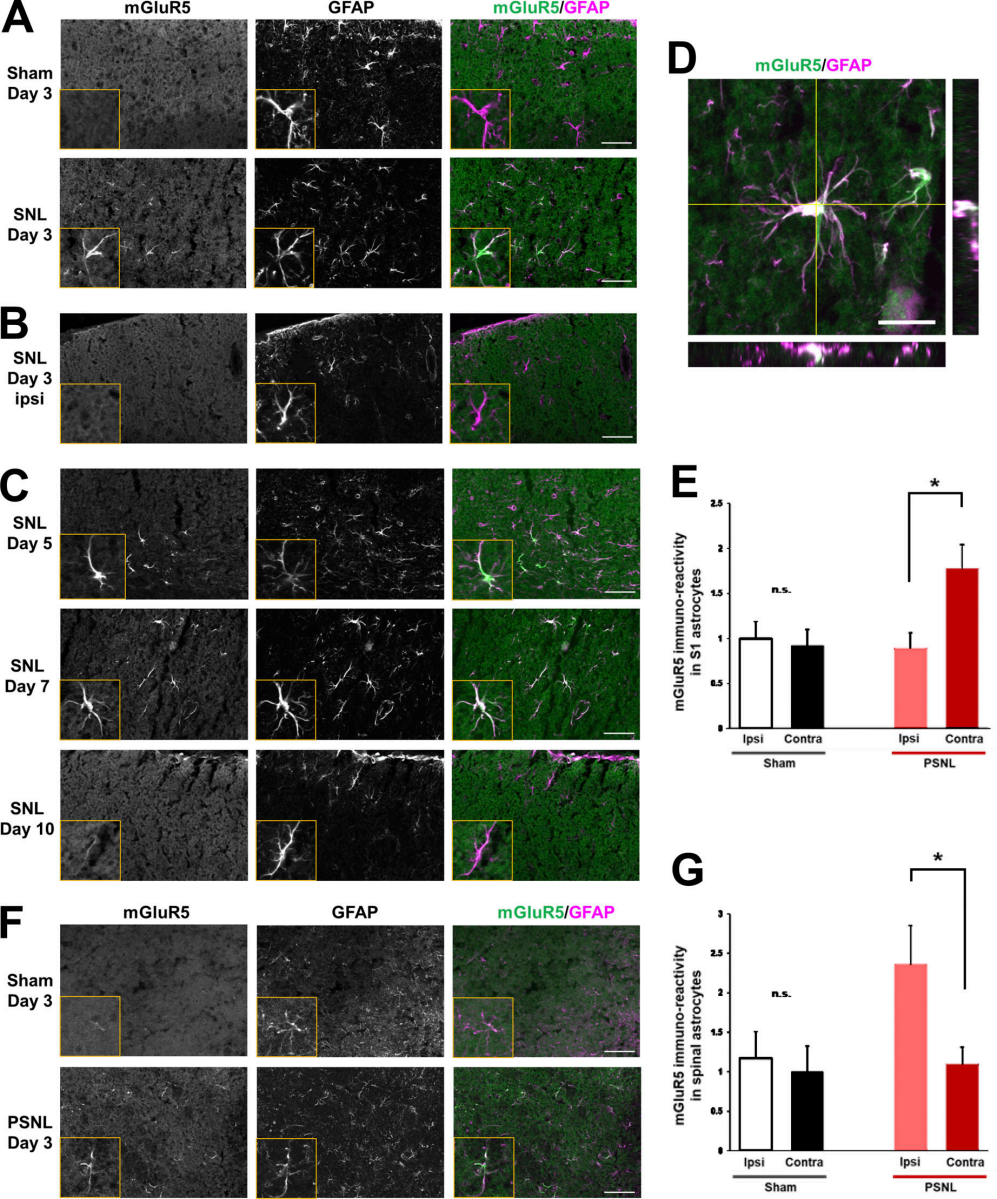
**Transient emergence of mGluR5 in S1 cortex astrocytes after PSNL. (A)** Immunohistochemical images of the contralateral S1 cortex (hindlimb region) in sham or PSNL-operated mice. Green indicates mGluR5, and magenta indicates GFAP. **(B)** Ipsilateral (ipsi) S1 cortex on day 3 after PSNL. **(C)** Immunohistochemical images of the contralateral (contra) S1 cortex on days 5, 7, and 10 after PSNL. **(D)** Immunohistochemical images of orthogonal projections of S1 astrocytes. **(E)** mGluR5 immunoreactivity of GFAP-positive cells in the S1 cortex on day 3 (106 cells quantified from seven mice comprised the ipsi-sham group, 113 cells quantified from seven mice comprised the contra-sham group, 75 cells quantified from seven mice comprised the ipsi-PSNL group, and 59 cells quantified from seven mice comprised the contra-PSNL group). P = 0.990 (ipsi vs. contra in the sham group) and P = 0.032 (ipsi vs. contra in the PSNL group) by two-way ANOVA followed by Tukey’s post hoc test. **(F)** Immunohistochemical images of ipsilateral lumbar vertebra 2 of the spinal cord 3 days after PSNL. **(G)** mGluR5 immunoreactivity of GFAP-positive cells in the spinal lumbar cord on day 3 (43 cells quantified from four mice comprised the ipsi-sham group, 45 cells quantified from four mice comprised the contra-sham group, 81 cells quantified from seven mice comprised the ipsi-PSNL group, and 79 cells quantified from seven mice comprised the contra-PSNL group). P = 0.994 (ipsi vs. contra in the sham group) and P = 0.047 (ipsi vs. contra in the PSNL group) by two-way ANOVA followed by Tukey’s post hoc test. Scale bar: 50 µm (A, B, C, and F); 20 µm (D). Data indicate means ± SEM. *, P < 0.05.

**Figure S1.**
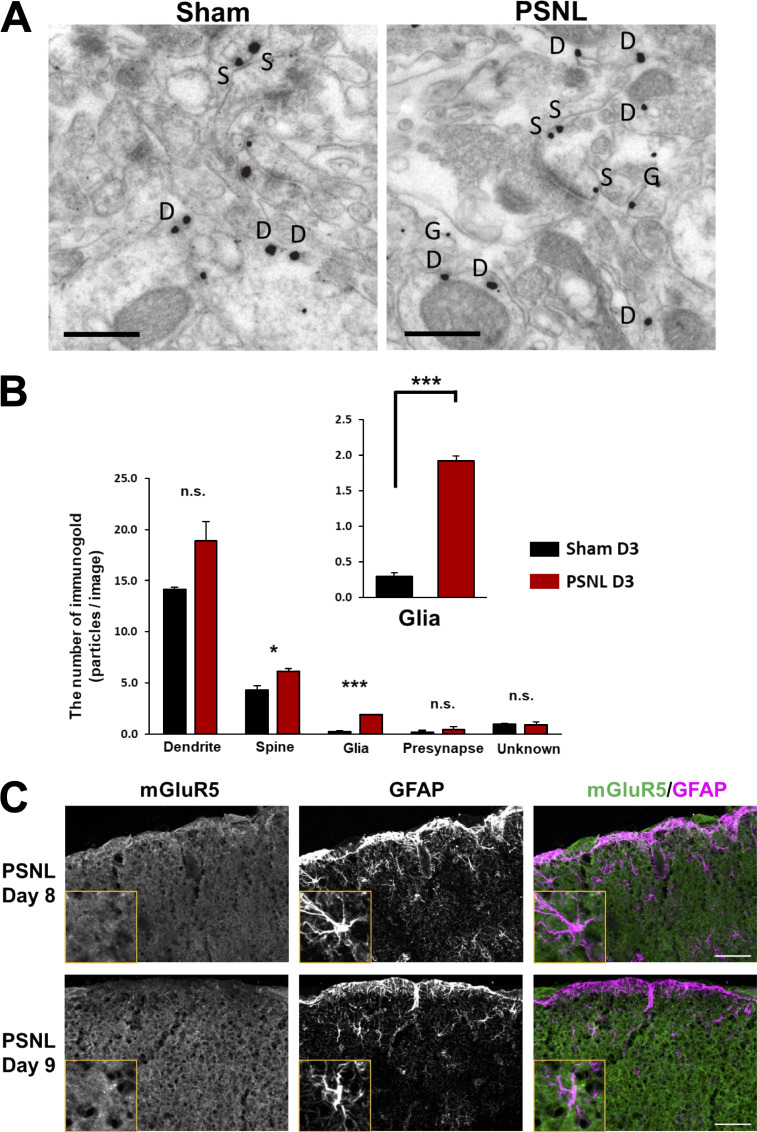
**Emergence of mGluR5 in S1 cortex astrocytes after PSNL. (A)** Representative electron micrographs of the contralateral S1 cortex labeled for mGluR5 from each group. Immunogold particles for mGluR5 were found in neuronal compartments and glial profiles, and distinct frequencies were found between sham and PSNL groups. D, dendrite; S, spine; G, glia. **(B)** The number of compartments with individual mGluR5 labeling in the sham (31 images in 3 mice) and PSNL (35 images in 3 mice) groups. The immunogold labeling profiles in each micrograph were classified as dendrites (sham = 280, PSNL = 348), spines (sham = 107, PSNL = 135), glia (sham = 9, PSNL = 51), presynaptic structures (axons, terminals; sham = 6, PSNL = 16), or unknown (sham = 28, PSNL = 32) on the basis of ultrastructural features. Particles/image: dendrites, 14.2 ± 0.22 vs. 18.9 ± 1.87 (sham vs. PSNL), P = 0.111; spines, 4.3 ± 0.41 vs. 6.2 ± 0.26 (sham vs. PSNL), P = 0.037; glia, 0.3 ± 0.05 vs. 1.9 ± 0.06 (sham vs. PSNL), P < 0.0001; presynaptic structures, 0.2 ± 0.20 vs. 0.5 ± 0.24 (sham vs. PSNL), P = 0.513; unknown, 1.0 ± 0.08 vs. 1.0 ± 0.25 (sham vs. PSNL), P = 0.969. **(C)** Immunohistochemical images of the contralateral S1 cortex (hindlimb region) on days 8 and 9 after PSNL. Scale bar: 0.5 µm (A); 50 µm (C). Data indicate means ± SEM. *, P < 0.05; ***, P < 0.001.

In astrocytes, the expression levels and function of mGluR5 are dynamically altered during development. Astrocytic mGluR5 is highly expressed in immature astrocytes and forms strong Ca^2+^ signals, but its expression and function decrease dramatically and disappear by a few weeks after birth (Cahoy et al., 2008; Rusnakova et al., 2013; Sun et al., 2013; Tanaka et al., 2021). However, it reemerges in astrocytes even in the adult brain, during some pathological conditions such as brain ischemia (Buscemi et al., 2017), Alzheimer’s disease (Grolla et al., 2013; Iyer et al., 2014; Shrivastava et al., 2013), Down’s syndrome (Iyer et al., 2014), amyotrophic lateral sclerosis (Anneser et al., 2004; Rossi et al., 2008), and epilepsy (Umpierre et al., 2016). First, we examined whether PSNL induces mGluR5 expression from the perspective of spatiotemporal patterns. In sham-operated mice, most astrocytes in the S1 cortex or the dorsal horn of the lumbar spinal cord did not express mGluR5 at any time after the surgery (Fig. 1 A). In PSNL-operated mice, GFAP (glial fibrillary acidic protein)-expressing astrocytes upregulated mGluR5 3 d after surgery (Figs. 1 A and S1 A). These findings were well associated with our previous functional data that mGluR5-mediated Ca^2+^ signals were absent in healthy adult S1 astrocytes but reappeared after PSNL (Kim et al., 2016). This increase was spatially restricted, i.e., in the contralateral S1 cortex of the hindlimb region (Fig. 1, A–E) and the ipsilateral spinal lumbar region (Fig. 1, F and G), which are areas associated with pain input in the cortex and spinal cord, respectively, but not in the ipsilateral S1 cortex (Fig. 1, B and E) or contralateral spinal cord (Fig. 1 G). The elevated mGluR5 signals in S1 astrocytes were sustained for ≥7 d after PSNL but were not observed after day 10 (Figs. 1 C and S1 B). mGluR5 upregulation exhibited very limited spatial (hindlimb region of the S1 cortex) and temporal (induction phase of pain) patterns, which coincided with intense PSNL-induced spine turnover, as we previously reported (Kim et al., 2016; Kim et al., 2011; Kim and Nabekura, 2011).

### Generation of astro-mGluR5-cKO mice

**Figure 2.**
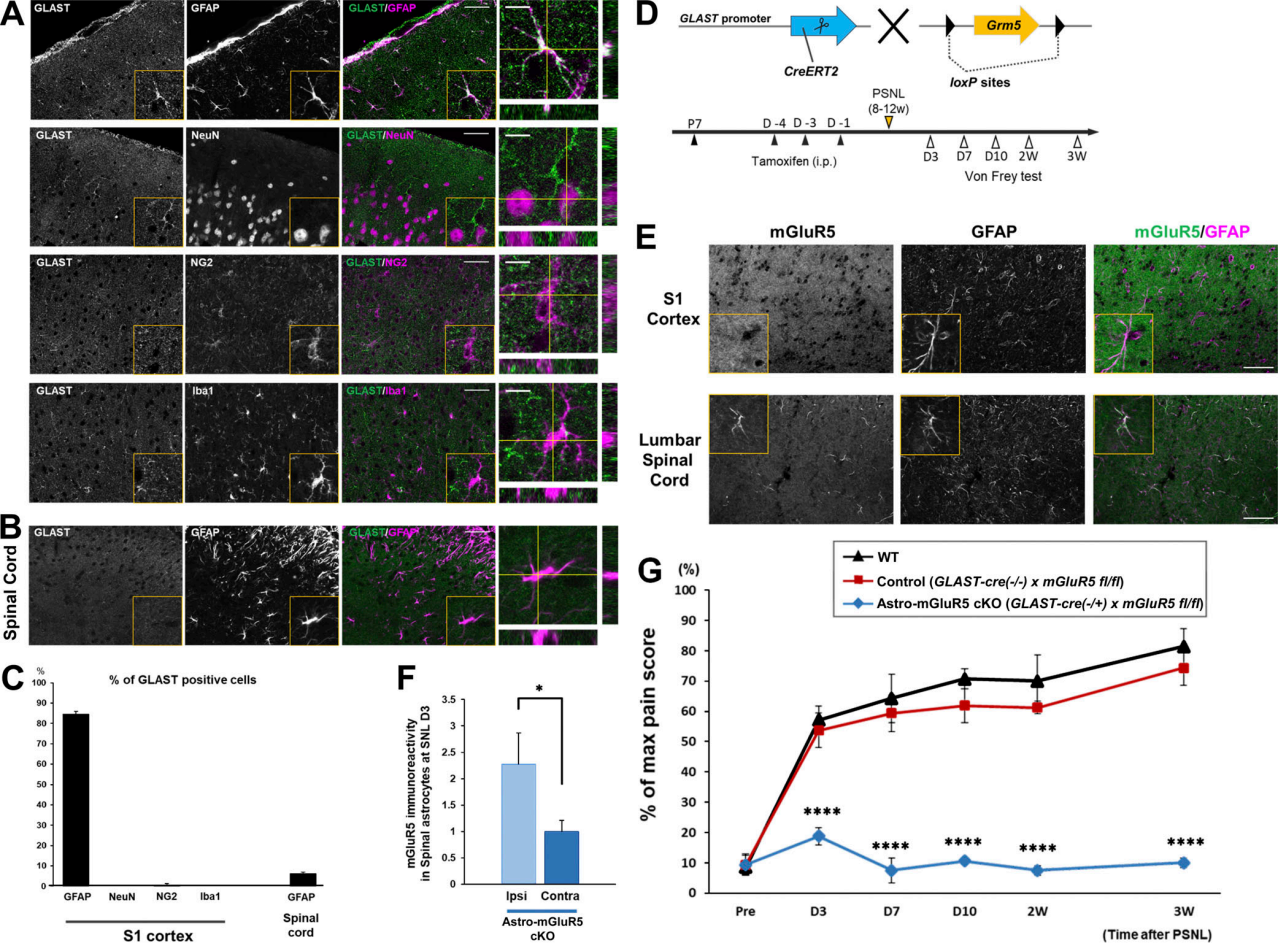
**Astrocytic mGluR5 in the S1 cortex is essential for the induction of neuropathic mechanical allodynia. (A and B)** Immunohistochemistry for GLAST in the S1 cortex (A) and lumbar vertebra 2 of the spinal cord (B). **(C)** Proportion of GLAST-expressing cells. 84.2% of GFAP-expressing cells (353/419 cells) were colabeled with GLAST, 0.00% of NenN (0/8,468 cells), 0.58% of NG2 (2/343 cells), 0.00% of Iba1 (0/513 cells), and 6.1% of spinal GFAP (26/428 cells). Immunohistochemical samples were obtained from three mice. **(D)** Gene trap strategy for astrocyte-specific mGluR5 deletion. **(E)** Immunohistochemical images of the contralateral S1 cortex in astro-mGluR5-cKO mice on day 3 after PSNL. **(F)** mGluR5 immunoreactivity of GFAP-positive cells in the spinal lumbar cord on day 3 in astro-mGluR5-cKO mice (31 cells quantified from five mice comprised the ipsilateral [ipsi] group, and 72 cells quantified from five mice comprised the contralateral [contra] group). P = 0.0497 by Welch’s *t* test. *, P < 0.05. **(G)** Time course showing the von Frey test score of WT (black, *n* = 7 mice), control (red, *n* = 8), and astro-mGluR5-cKO mice (blue, *n* = 8). Control vs. astro-mGluR5-cKO mice; P = 0.994 (pre); ****, P < 0.0001 (days 3, 7, and 10 and weeks 2 and 3), by two-way repeated-measures ANOVA. Scale bar: 50 µm (A, B, and E). Orthogonal projections of scale bar: 10 µm (A and B). Data indicate means ± SEM.

To determine whether astrocytic mGluR5 in the S1 cortex is required for the induction of synapse remodeling and neuropathic pain, astro-mGluR5-cKO mice were generated. GLAST (glutamate aspartate transporter 1), encoded by the *Slc1a3* gene, is reported to be highly expressed in cerebral cortical astrocytes but not in spinal cord astrocytes (Borggrewe et al., 2021; Regan et al., 2007). Therefore, we first investigated expression patterns of GLAST in the brain and spinal astrocytes by immunohistochemistry. As shown in Fig. 2, A–C, and Fig. S1, astrocytes showed GLAST-positive signals in the S1 cortex, whereas these signals were not observed in spinal cord astrocytes. Thus, to achieve brain astrocyte–specific mGluR5 deletion, we crossed GLAST-CreERT2 mice with *Grm5*^*flx/flx*^ mice (Fig. 2 D). In astro-mGluR5-cKO mice administered tamoxifen, the PSNL-induced upregulation of astrocytic mGluR5 in S1 cortical astrocytes (Fig. 2 E) was completely abolished, whereas that in ipsilateral lumbar spinal cord astrocytes was not reduced (Fig. 2, E and F). These results indicate that GLAST promotor-mediated deletion of *Grm5* in S1 astrocytes can be used to specifically delete mGluR5 in cortical astrocytes without affecting spinal cord astrocytes.

### Astrocytic mGluR5 in the S1 cortex is essential for the induction of neuropathic mechanical allodynia

Next, we investigated whether astrocytic mGluR5 was required for the pathogenesis of neuropathic pain caused by PSNL. We performed PSNL surgery on astro-mGluR5-cKO, GLAST-CreERT2^−/−^::*Grm5*^*flx/flx*^ (negative control littermates), and WT mice and measured mechanical allodynia using the von Frey test (Fig. 2 G). In WT and control littermate mice, PSNL-induced mechanical allodynia was observed 3 d after surgery and lasted for ≥3 wk. In contrast, PSNL-induced mechanical allodynia was never observed in astro-mGluR5-cKO mice (Fig. 2 G). These findings demonstrated that although the reappearance of mGluR5 was transient and restricted in S1 astrocytes, this limited event is essential for the development of mechanical allodynia.

### Activation of mGluR5 is responsible for Ca^2+^ events in S1 astrocytes

**Figure 3.**
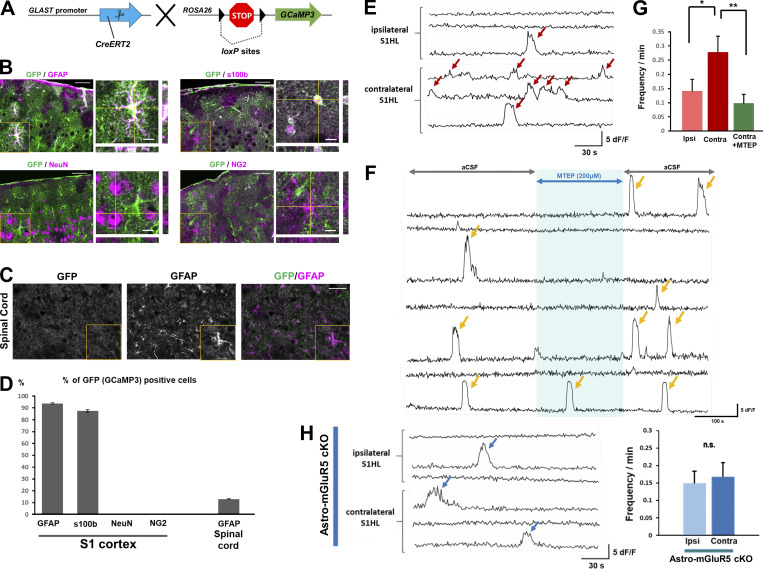
**Activation of mGluR5 is responsible for Ca**^**2+**^ **events in S1 cortex astrocytes. (A)** Gene trap strategy used to insert conditional *GCaMP3* into the *ROSA26* gene. **(B and C)** *GCaMP3* coding mice stained for GFP (GCaMP3) and cell-specific markers in the S1 cortex (B) and spinal cord (C). **(D)** Proportion of GFP (GCaMP3)-expressing cells. 93.6% of GFAP-expressing cells (1,047/1,119 cells) were colabeled with GFP, 87.3% of S100β (1,883/2,157 cells), 0.00% of NenN (0/6,591 cells), 0.82% of NG2 (9/1,100 cells), and 12.9% of spinal GFAP (156/1,208 cells). Immunohistochemical samples were obtained from three mice. **(E and F)** Representative traces of Ca^2+^ fluctuations measured in astrocytes on day 3 after PSNL (E) and under MTEP (200 µM) application (F). **(G)** The graph shows the mean Ca^2+^ fluctuation frequency. P = 0.028 (ipsilateral [ipsi] group vs. contralateral [contra] group) and P = 0.003 (contra vs. contra + MTEP) by one-way ANOVA followed by Fisher’s post hoc test. 36 cells quantified from three mice comprised the ipsi group, and 30 cells quantified from three mice comprised the contra group. **(H)** Representative traces of Ca^2+^ fluctuations in astro-mGluR5-cKO mice measured in astrocytes on day 3 after PSNL. The graph shows the mean Ca^2+^ fluctuation frequency (P = 0.726) in astro-mGluR5-cKO mice. 46 cells quantified from three mice comprised the ipsi group, and 32 cells quantified from three mice comprised the contra group. Scale bar: 50 µm (B and C). Orthogonal projections of scale bar: 10 µm (B). Data indicate means ± SEM. *, P < 0.05; **, P < 0.01.

**Figure S2.**
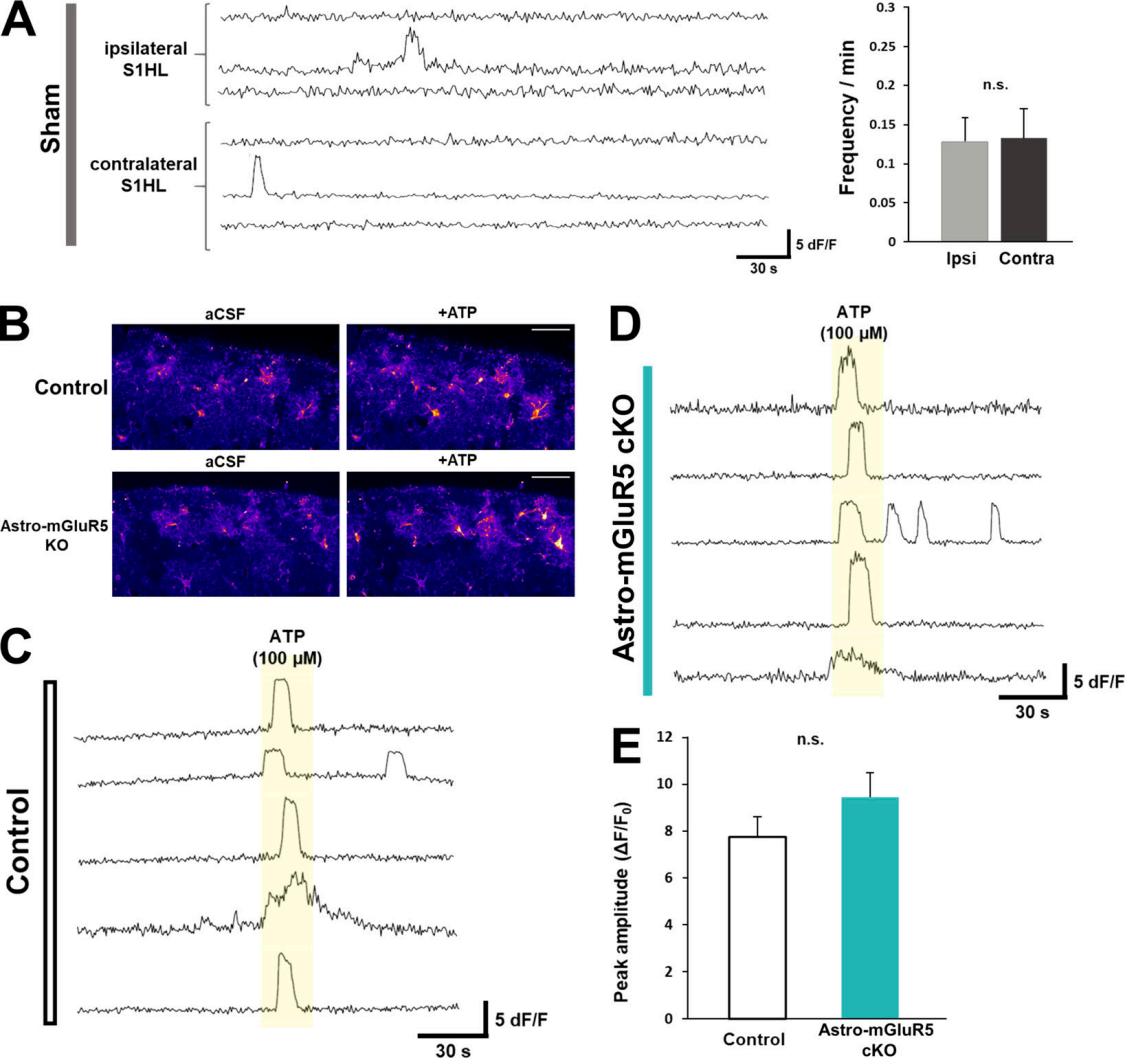
**Activation of mGluR5 is responsible for Ca**^**2+**^ **events in S1 cortex astrocytes. (A)** Representative traces of Ca^2+^ fluctuations in sham-operated mice measured in astrocytes on day 3 after PSNL. The graph shows the mean Ca^2+^ fluctuation frequency (P = 0.839). 42 cells quantified from 3 mice comprised the ipsilateral (ipsi) group, and 38 cells quantified from 3 mice comprised the contralateral (contra) group. **(B)** Representative GCaMP3 signals illustrate the Ca^2+^ response to ATP application. **(C and D)** Representative traces showing fluctuations of ATP-evoked Ca^2+^ transients in control mice (C) and astro-mGluR5 cKO mice (D). **(E)** The graph shows the ATP-evoked amplitude (Δ*F*/*F*_0_, P = 0.839). 19 cells were quantified from 3 control mice, and 28 cells were quantified from 4 astro-mGluR5-cKO mice. Scale bar: 50 µm (B). Data indicate means ± SEM.

As we have previously shown that increased intracellular Ca^2+^ signals in S1 astrocytes play a key role in the pathogenesis of neuropathic pain, we next examined whether astrocytic mGluR5 was responsible for the increase in Ca^2+^ signaling. To visualize astrocytic Ca^2+^ signals, we crossed GLAST-Cre/ERT2 mice with mice harboring a *CAG-loxp-stop-loxp-GCaMP3* cassette in the *ROSA26* locus (Fig. 3 A). We initially examined Cre/ERT2-mediated expression of GCaMP3 by immunohistochemistry, followed by tamoxifen administration (Fig. 3, B–D). We observed that GCaMP3-expressing cells were colocalized with GFAP and S100β (astrocytic marker) but not with NeuN or NG2 (Fig. 3 B). We generated GCaMP3-expressing astro-mGluR5-cKO mice by crossing GLAST-Cre/ERT2 mice with *Grm5*^*flx/flx*^ mice to study astrocyte Ca^2+^ signals in acute brain slices of the S1 cortex of astro-mGluR5-cKO mice. Consistent with our previous results obtained by in vivo Ca^2+^ imaging methods (Kim et al., 2016), we found that PSNL significantly increased the frequency of spontaneous Ca^2+^ fluctuations in contralateral S1 astrocytes of layer I on day 3 (Fig. 3 E) compared with ipsilateral S1 astrocytes. These frequent Ca^2+^ fluctuations were decreased by administration of the potent and selective mGluR5 antagonist, 3-[2-(2-methyl-4-thiazolyl)ethynyl]pyridine (MTEP; Fig. 3, F and G). These frequent Ca^2+^ fluctuations were not observed in astro-mGluR5-cKO mice (Fig. 3 H) or sham-operated mice (Fig. S2 A). Furthermore, there was no significant difference in the frequency of Ca^2+^ fluctuations between contralateral and ipsilateral S1 astrocytes in PSNL-treated astro-mGluR5-cKO mice. Because PSNL can increase Ca^2+^ fluctuations, even in ipsilateral cortical astrocytes in vivo (Ishikawa et al., 2018), we also compared the Ca^2+^ signals in ipsilateral S1 astrocytes between PSNL-treated astro-mGluR5-cKO and sham-operated WT mice and found no significant differences (PSNL-treated astro-mGluR5-cKO mice 0.17 ± 0.040, *n* = 32, vs. sham-operated WT mice 0.13 ± 0.037, *n* = 38; P = 0.59). Astrocytes in control mice and astro-mGluR5-cKO mice similarly responded to ATP stimulation (Fig. S2, B–E). Therefore, we concluded that the enhanced Ca^2+^ fluctuations in S1 astrocytes induced by PSNL were caused by the transient reemergence of mGluR5.

### Upregulation of an mGluR5-dependent synaptogenic molecule, TSP1, in S1 astrocytes

**Figure 4.**
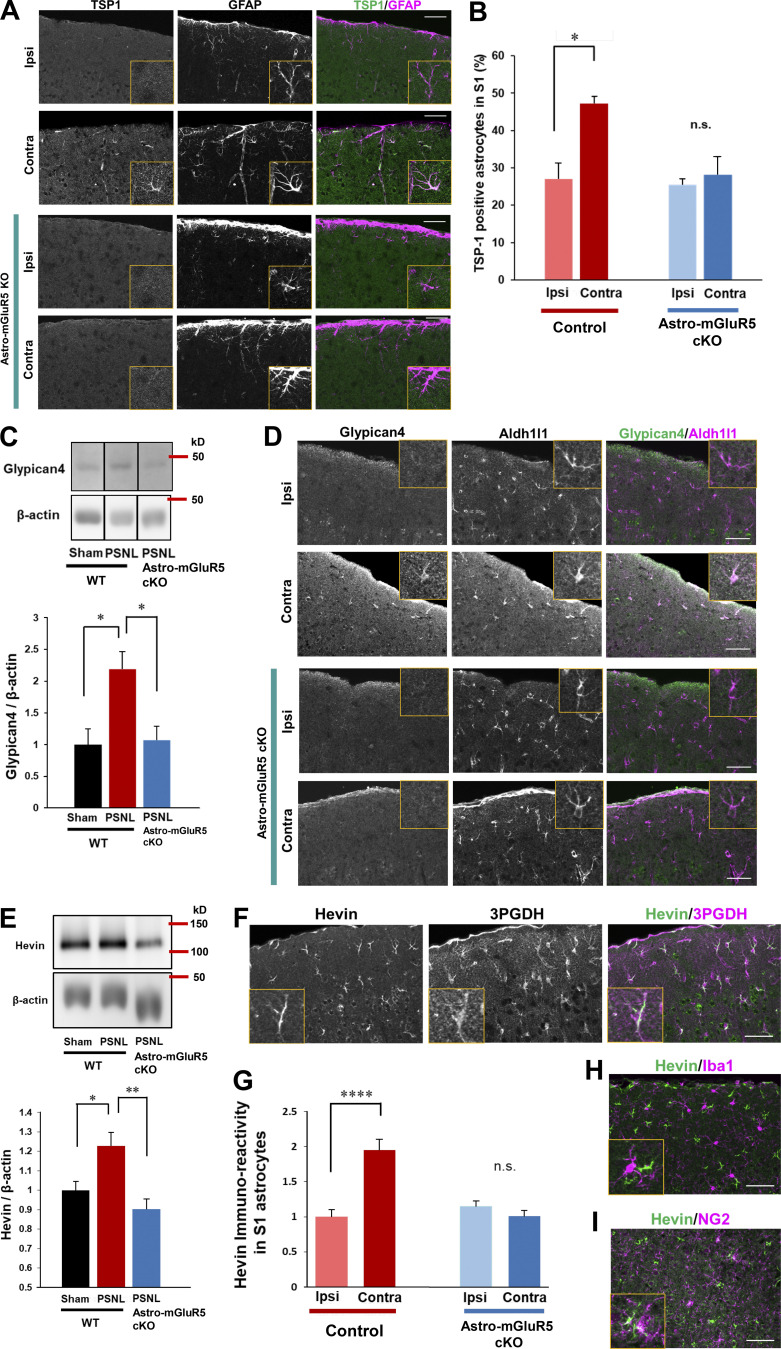
**Astrocytic mGluR5 drives multiple synaptogenic molecules in the S1 cortex after PSNL. (A)** Immunohistochemical staining of TSP1 and GFAP in the S1 cortex on day 3 after PSNL. **(B)** Proportion of TSP1-expressing S1 astrocytes on day 3 after PSNL. Control mice; ipsilateral (ipsi) vs. contralateral (contra) group, P = 0.014; astro-mGluR5-cKO mice; ipsi vs. contra, P = 0.947, by two-way ANOVA followed by Tukey’s post hoc test. *n* = 3 mice per group. **(C)** Western blotting of Glypican-4 expression in contralateral S1 cortical samples on day 3 after PSNL. P = 0.023 (sham-WT vs. PSNL-WT) and P = 0.019 (PSNL-WT vs. PSNL-astro-mGluR5-cKO) by one-way ANOVA followed by Tukey’s post hoc test. *n* = 4 mice (sham-WT), 4 mice (PSNL-WT), and 6 mice (PSNL-astro-mGluR5-cKO). **(D)** Immunohistochemical images of Glypican-4 in S1 astrocytes on day 3 after PSNL. **(E)** Western blotting of Hevin expression in contralateral S1 cortical samples on day 3 after PSNL. P = 0.029 (sham-WT vs. PSNL-WT) and P = 0.0032 (PSNL-WT vs. PSNL-astro-mGluR5-cKO) by one-way ANOVA followed by Tukey’s post hoc test. *n* = 7 mice (sham-WT), 7 mice (PSNL-WT), and 6 mice (PSNL-astro-mGluR5-cKO). **(F)** Immunohistochemical staining of Hevin and 3PGDH (astrocytic marker). **(G)** Hevin immunoreactivity in S1 astrocytes on day 3 (41 cells quantified from three mice comprised the ipsi-control group, 45 cells quantified from three mice comprised the contra-control group, 46 cells quantified from three mice comprised the ipsi-astro-mGluR5-cKO group, and 52 cells quantified from three mice comprised the contra-astro-mGluR5-cKO group). P < 0.0001 (ipsi vs. contra in the control group) and P = 0.768 (ipsi vs. contra in the sham group) by two-way ANOVA followed by Tukey’s post hoc test. **(H and I)** Immunohistochemical staining of Hevin and Iba1 (microglial marker; H) and NG2 (oligodendrocyte precursor cell marker; I). Scale bar: 50 µm. Data indicate means ± SEM. *, P < 0.05; **, P < 0.01; ****, P < 0.0001. Source data are available for this figure: SourceData F4.

Previously, we found that TSP1, a synaptogenic molecule induced by Ca^2+^ signaling in astrocytes, is required for the induction of synapse remodeling and mechanical allodynia (Kim et al., 2016; Kim et al., 2017). Thus, we determined whether astrocytic mGluR5 is required for PSNL-induced TSP1 production using astro-mGluR5-cKO mice. Consistent with our previous reports, PSNL significantly upregulated TSP1 on the contralateral side of the S1 cortex in WT mice, which was colocalized with GFAP-positive astrocytes (Fig. 4 A). However, TSP1-positive astrocytes were not increased in astro-mGluR5-cKO mice after PSNL (Fig. 4, A and B). Therefore, we concluded that PSNL-induced TSP1 expression in S1 astrocytes was dependent on astrocytic mGluR5.

### Astrocytic mGluR5 drives Glypican-4 and Hevin in induction of mechanical allodynia

In addition to TSP1, astrocytes produce multiple synaptogenic molecules. Thus, we investigated whether PSNL induces other synaptogenic molecules such as Glypican-4 and Hevin in S1 cortical astrocytes after PSNL. By Western blotting, we found that Glypican-4 was also increased in the S1 cortex after PSNL, and this increase was absent in astro-mGluR5-cKO mice (Fig. 4 C). Immunohistochemical analysis showed that Glypican-4–positive signals in the contralateral S1 cortex on day 3 were colocalized with Aldh1l1, an astrocyte-specific marker (Fig. 4 D). Again, this increase was not observed in astro-mGluR5-cKO mice (Fig. 4 D).

**Figure S3.**
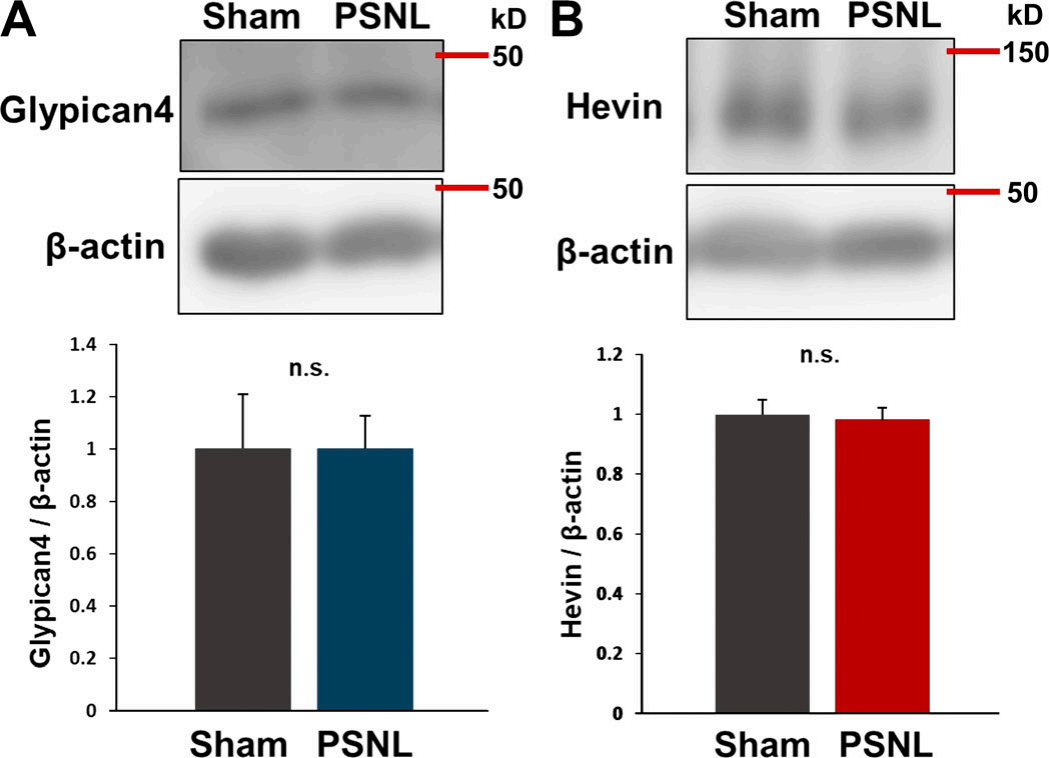
**Increases in Glypican-4 or Hevin in the S1 cortex were not observed on day 15 after PSNL. (A and B)** Western blotting of Glypican-4 (A) and Hevin expression (B) in contralateral S1 cortical samples on day 15 after PSNL. Four mice per group were analyzed. P = 0.999 (Glypican-4; sham vs. PSNL) and P = 0.826 (Hevin; sham vs. PSNL) via *t* test. Data indicate means ± SEM. Source data are available for this figure: SourceData FS3.

We also found that PSNL increased Hevin, another synaptogenic molecule that connects thalamic axons and cortical dendrites (Risher et al., 2014; Singh et al., 2016). Western blotting showed that PSNL upregulated Hevin in WT but not in astro-mGluR5-cKO mice (Fig. 4 E). Similarly, immunohistochemical analysis revealed that PSNL upregulated Hevin in contralateral S1 astrocytes (3PDGH positive) in WT mice but not in astro-mGluR5-cKO mice (Fig. 4, F and G). Hevin was not expressed by microglia (Fig. 4 H) or oligodendrocyte precursor cells in the S1 cortex (Fig. 4 I). The upregulation of Glypican-4 and Hevin in the S1 cortex was decreased on day 15 after PSNL (Fig. S3).

**Figure 5.**
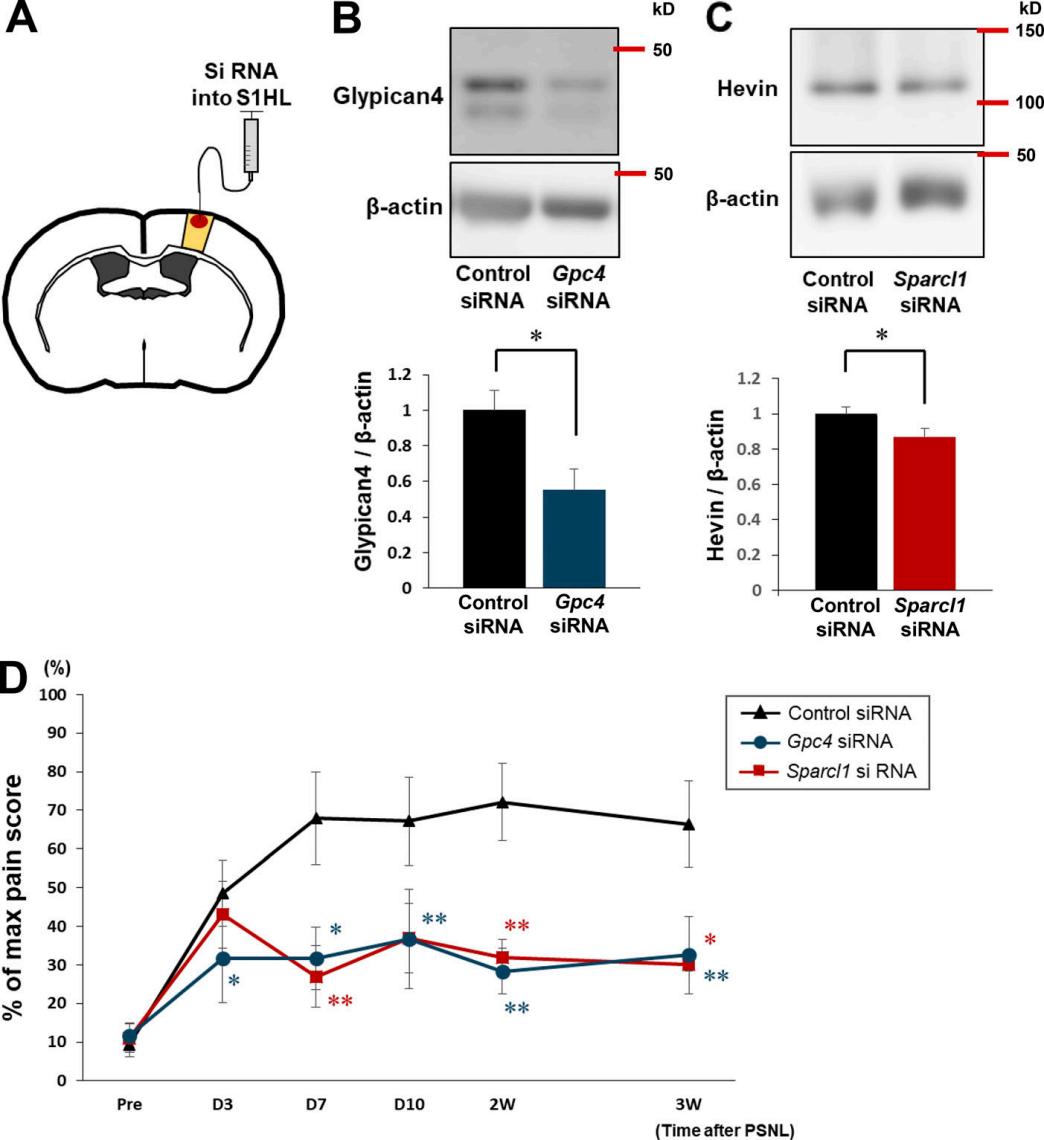
**Upregulation of Glypican-4 and Hevin is required for the induction of mechanical allodynia. (A)** Schematic illustration showing the knockdown of *Gpc4* and *Sparcl1* in the S1 cortex by siRNA. **(B and C)** Western blotting of Glypican-4 (B) and Hevin (C), showing the effect of siRNA administration in the contralateral S1 cortex on day 3 after PSNL. P = 0.0498 (control siRNA vs. *Gpc4* siRNA) and P = 0.0477 (control siRNA vs. *Sparcl1* siRNA). **(D)** Time course showing the von Frey test score of control mice administered siRNA (black, *n* = 7), *Gpc4* mice administered siRNA (blue, *n* = 6), and *Sparcl1* mice administered siRNA (red, *n* = 5). Control siRNA vs. *Gpc4* siRNA; P = 0.0195 (day 3), P = 0.0405 (day 7), P = 0.0089 (day 10), P = 0.0022 (2 wk), and P = 0.0022 (3 wk); Control siRNA vs. *Sparcl1* siRNA; P = 0.752 (day 3), P = 0.0038 (day 7), P = 0.051 (day 10), P = 0.0038 (2 wk), P = 0.012 (3 wk); by two-way repeated-measures ANOVA. Data indicate means ± SEM. *, P < 0.05; **, P < 0.01. Source data are available for this figure: SourceData F5.

We next investigated whether the increases in Glypican-4 or Hevin in the S1 cortex are also required for the induction of neuropathic pain and mechanical allodynia caused by PSNL. We injected siRNAs into the contralateral S1 cortex immediately before PSNL (Fig. 5 A) to knock down *Gpc4* (encoding Glypican-4) or *Sparcl1* (encoding Hevin; Fig. 5, B and C). As shown in Fig. 5 D, PSNL-evoked mechanical allodynia was attenuated by either *Gpc4* siRNA or *Sparcl1* siRNA. This inhibition lasted for ≥3 wk, with a time course similar to that in astro-mGluR5-cKO mice. Therefore, these synaptogenic molecules are also involved in the pathology of mechanical allodynia, especially in the induction phase. We concluded that the upregulation of mGluR5 by PSNL controls multiple synaptogenic molecules including TSP1, Glypican-4, and Hevin, suggesting that reemergence of mGluR5 in S1 cortical astrocytes would function as an organizer of S1 cortical networks, leading to the misconnection of networks and mechanical allodynia. In addition, the reemergence of astrocytic mGluR5 might be a critical event that controls synapse plasticity in the adult brain.

### Astrocytic mGluR5 induced excessive synapse formation and enhanced neuronal activity

**Figure 6.**
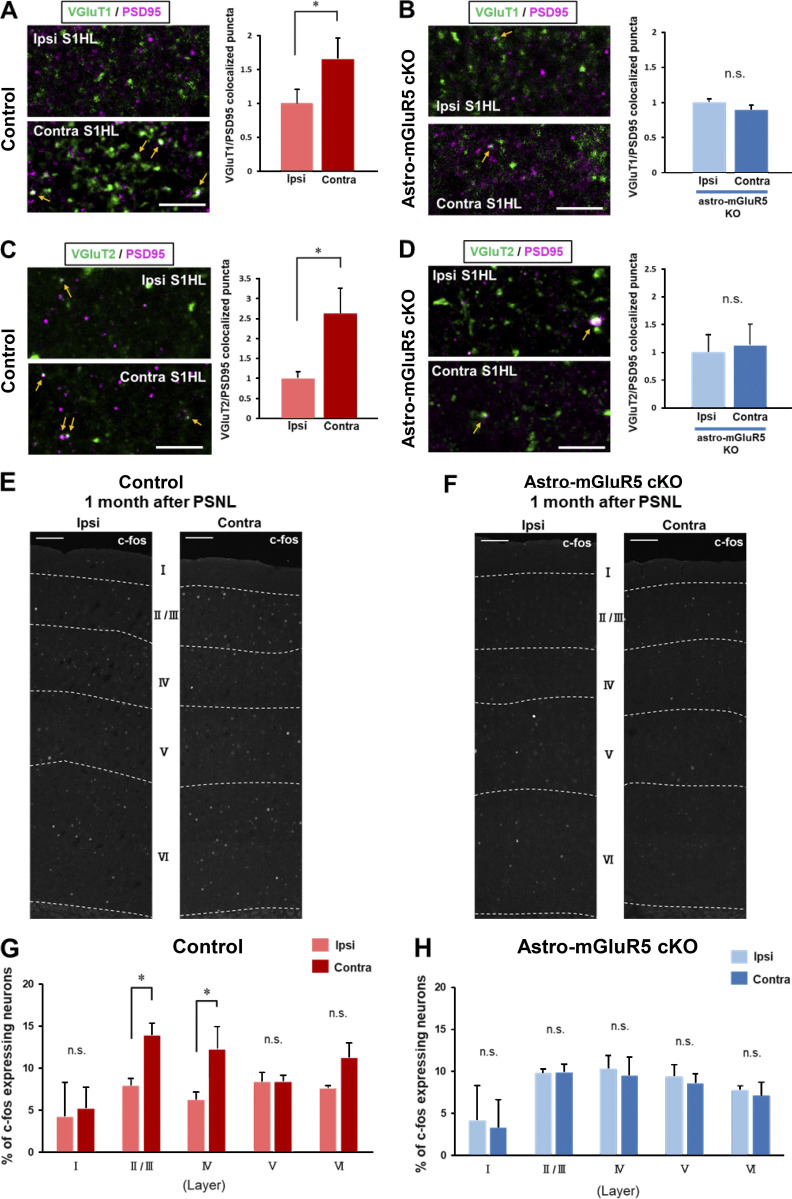
**Astrocytic mGluR5 induced excessive synapse formation and enhanced neuronal activity. (A)** Immunohistochemistry for VGluT1 (green) and PSD95 (magenta) in the ipsilateral (ipsi) and contralateral (contra) S1 cortex on day 3 after PSNL. **(B)** Immunohistochemical staining in the ipsilateral and contralateral S1 cortex of astro-mGluR5-cKO mice on day 3 after PSNL. **(A and B) **Yellow arrows show colocalized VGluT1 and PSD95 signals. Right bars show the quantitative analysis of colocalized VGluT1 and PSD95 puncta on day 3 in control WT (three mice/group, P = 0.0231) and astro-mGluR5-cKO mice (three mice/group, P = 0.196). Data are normalized by the synaptic number (colocalized signals) of the ipsilateral S1 cortex. **(C and D)** Immunohistochemical staining of VGluT2 (green) and PSD95 (magenta) in the contralateral S1 cortex of the control WT group on day 3 after PSNL (C) and in astro-mGluR5-cKO mice (D). **(C and D) **Yellow arrows show colocalized VGluT2 and PSD95 signals. The bars on the right show the quantitative analysis of VGluT2 and PSD95 double-positive signals on day 3 in control WT (4 mice/group, P = 0.0488) and astro-mGluR5-cKO mice (4 mice/group, P = 0.4598). **(E and F)** Representative confocal image of c-fos in the S1 cortex of control mice, 1 month after PSNL (E), and in the S1 cortex of astro-mGluR5-cKO mice (F). **(G and H)** The proportion of c-fos–expressing NeuN-positive neurons in the control group 1 month after PSNL (3 mice/group). Layer I; P = 0.732, II/III; P = 0.0482, IV; P = 0.0479, V; P = 0.986, VI; P = 0.222; G) and in the astro-mGluR5-cKO group (3 mice/group. Layer I; P = 0.780, II/III; P = 0.975, IV; P = 0.784, V; P = 0.771, VI; P = 0.835; H) by two-way ANOVA followed by Fisher’s post hoc test. Scale bars: 5 µm (A–D); 100 µm (E and F). Data indicate means ± SEM. *, P < 0.05.

We have shown that transient astrocytic mGluR5 reemergence induces multiple synaptogenic molecules after PSNL, which may facilitate synapse remodeling in S1 circuits by forming excitatory synapses. Thus, we next quantified excitatory synapses according to the colocalization of the presynaptic markers VGlut1 (intracortical synapses) or VGlut2 (thalamocortical synapses) with the postsynaptic marker PSD95 (Kaneko and Fujiyama, 2002). 3 d after PSNL, the ipsi- and contralateral S1 cortex was double-stained for PSD95 and VGlut1 or VGlut2. PSNL increased the colocalized puncta of VGlut1/PSD95 in layer I (i.e., synaptic zone) of the contralateral S1 cortex (Fig. 6 A) in WT mice but not in astro-mGluR5-cKO mice (Fig. 6 B). We also detected a PSNL-induced increase in VGlut2/PSD95 colocalized puncta in control WT mice (Fig. 6 C) but not in astro-mGluR5-cKO mice (Fig. 6 D). Therefore, we concluded that the increase in excitatory synapse remodeling in the S1 cortex induced by PSNL, which we previously demonstrated (Kim et al., 2016; Kim et al., 2011), was dependent on astrocytic mGluR5. In addition, we further demonstrated that astrocytic mGluR5 is essential for both intracortical and thalamocortical synapses after PSNL.

Our results showed that astrocytic mGluR5 reemergence induces multiple synaptogenic molecules after PSNL, leading to excessive excitatory synapse formation. This event was transient; however, it causes long-lasting mechanical allodynia. Thus, we investigated whether PSNL caused sustained changes in neuronal activity in the S1 cortex by examining the proportion of neurons that expressed c-fos–positive signals (a marker of recent neuronal activity) after PSNL. Even 1 mo after PSNL, we found a significantly higher proportion of c-fos–expressing neurons in layers II/III and IV of the contralateral S1 cortex than in the ipsilateral side (Fig. 6, E and G) in WT mice. Although a similar tendency was observed in layer VI, statistical significance was not reached. This increase in PSNL-evoked c-fos–positive neurons in layers II/III in WT mice was abolished in astro-mGluR5-cKO mice (Fig. 6, F and H). These results indicate that astrocytic mGluR5 is essential for increased neuronal activity in the S1 cortex, especially in layers II/III, which was sustained for ≥1 mo after PSNL, although the reemergence of mGluR5 was transient (∼1 wk).

### Astrocytic mGluR5 induced excessive synapse formation and enhanced neuronal activity

**Figure 7.**
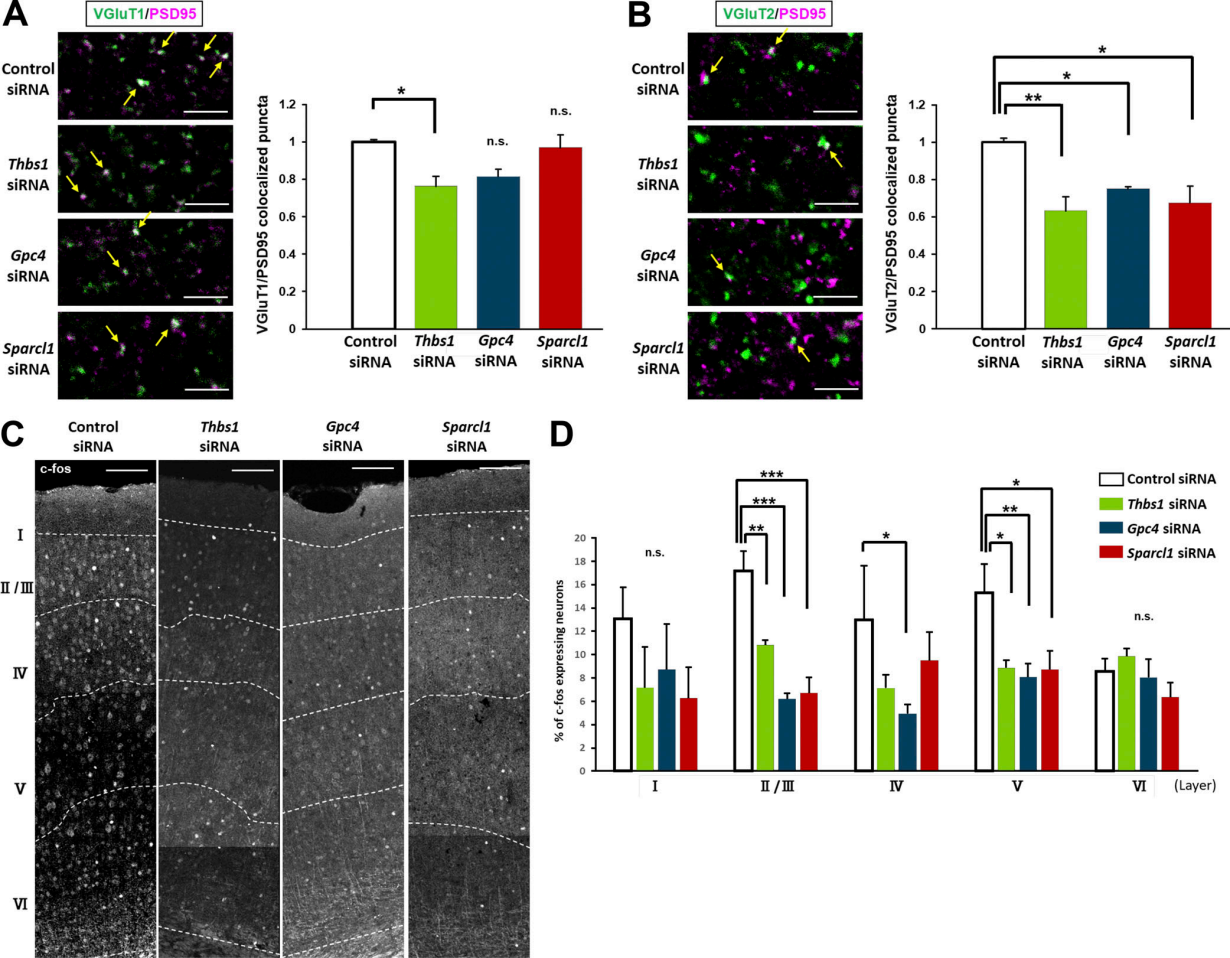
**Knockdown of astrocytic synaptogenic molecules suppressed excessive synapse formation and enhanced neuronal activity. (A)** Immunohistochemical analysis of VGluT1 (green) and PSD95 (magenta) in the contralateral S1 cortex on day 3 after PSNL. Yellow arrows show colocalized VGluT1 and PSD95 signals. The bar graph on the right shows the quantitative analysis of colocalized VGluT1 and PSD95 puncta on day 3. P = 0.020 (control siRNA vs. *Thbs1* siRNA), P = 0.056 (control siRNA vs. *GPC4* siRNA) and P = 0.78 (control siRNA vs. *Sparcl1* siRNA). Data are normalized by the number of synapses (colocalized signals) in the control siRNA group. **(B)** Immunohistochemical staining of VGluT2 (green) and PSD95 (magenta) in the contralateral S1 cortex. The bar graph on the right shows the quantitative analysis of VGluT2 and PSD95 double-positive signals on day 3. P = 0.0066 (control siRNA vs. *Thbs1* siRNA), P = 0.044 (control siRNA vs. *GPC4* siRNA), and P = 0.013 (control siRNA vs. *Sparcl1* siRNA). Data are normalized to the number of synapses (colocalized signals) in the control siRNA group. **(A and B)** Yellow arrows show colocalized VGluT2 and PSD95 signals. **(C)** Confocal image of c-fos in the contralateral S1 cortex 1 month after PSNL. **(D)** The proportion of c-fos–expressing NeuN-positive neurons in the contralateral S1 cortex at 1 mo after PSNL (four mice in the control siRNA group and the *Thbs1* siRNA group, five mice in the *Gpc4* siRNA group and *Sparcl1* siRNA group). Layer I; P = 0.822 (control vs. *Thbs1* siRNA), P = 0.937 (control vs. *Gpc4* siRNA), P = 0.667 (control vs. *Sparcl1* siRNA). Layer II/III; P = 0.0016 (control vs. *Thbs1* siRNA), P < 0.0001 (control vs. *Gpc4* siRNA), P < 0.0001 (control vs. *Sparcl1* siRNA). Layer IV; P = 0.149 (control vs. *Thbs1* siRNA), P = 0.044 (control vs. *Gpc4* siRNA), P = 0.354 (control vs. *Sparcl1* siRNA). Layer V; P = 0.0156 (control vs. *Thbs1* siRNA), P = 0.0058 (control vs. *Gpc4* siRNA), P = 0.0104 (control vs. *Sparcl1* siRNA). Layer VI; P = 0.499 (control vs. *Thbs1* siRNA), P = 0.783 (control vs. *Gpc4* siRNA), P = 0.248 (control vs. *Sparcl1* siRNA) by one-way ANOVA followed by Fisher’s post hoc test. Scale bars: 5 µm (A and B); 100 µm (C). Data indicate means ± SEM. *, P < 0.05; **, P < 0.01; ***, P < 0.001.

We have shown that astrocytic mGluR5 drives multiple synaptogenic molecules after PSNL, which is important for the pathogenesis of mechanical allodynia. Thus, we investigated whether TSP1, Glypican-4, and Hevin are required for excitatory synapse formation. We knocked down these genes in the contralateral S1 cortex and quantified the colocalization of presynaptic VGlut1 or VGlut2 with postsynaptic PSD95 on day 3 after PSNL. After knocking down *Thbs1*, the number of colocalized puncta of both VGluT1/PSD95 and VGluT2/PSD95 were significantly decreased on day 3 (Fig. 7, A and B). However, knockdown of *Gpc4* or *Sparcl1* did not suppress the number of VGluT1-positive synapses compared with the control group (Fig. 7 A). In contrast, the number of colocalized VGluT2 and PSD95 puncta was significantly decreased, even in the *Gpc4* and *Sparcl1* knockdown groups (Fig. 7 B). Finally, we investigated whether long-lasting PSNL-induced changes in neuronal activity are involved in upregulation of these synaptogenic molecules. We quantified c-fos–positive signals 1 mo after PSNL in the S1 cortex of *Thbs1*, *Gpc4*, or *Sparcl1* knockdown mice. As shown in Fig. 7 D, the proportion of c-fos–expressing neurons was decreased, especially in layer II/III and V in the *Thbs1*, *Gpc4*, and *Sparcl1* knockdown groups. Thus, we conclude that TSP1, Glypican-4, and Hevin expression are indeed responsible for the changes in astrocytic mGluR5-triggered synapse formation and enhanced neuronal activity shown in Fig. 6.

**Figure 8.**
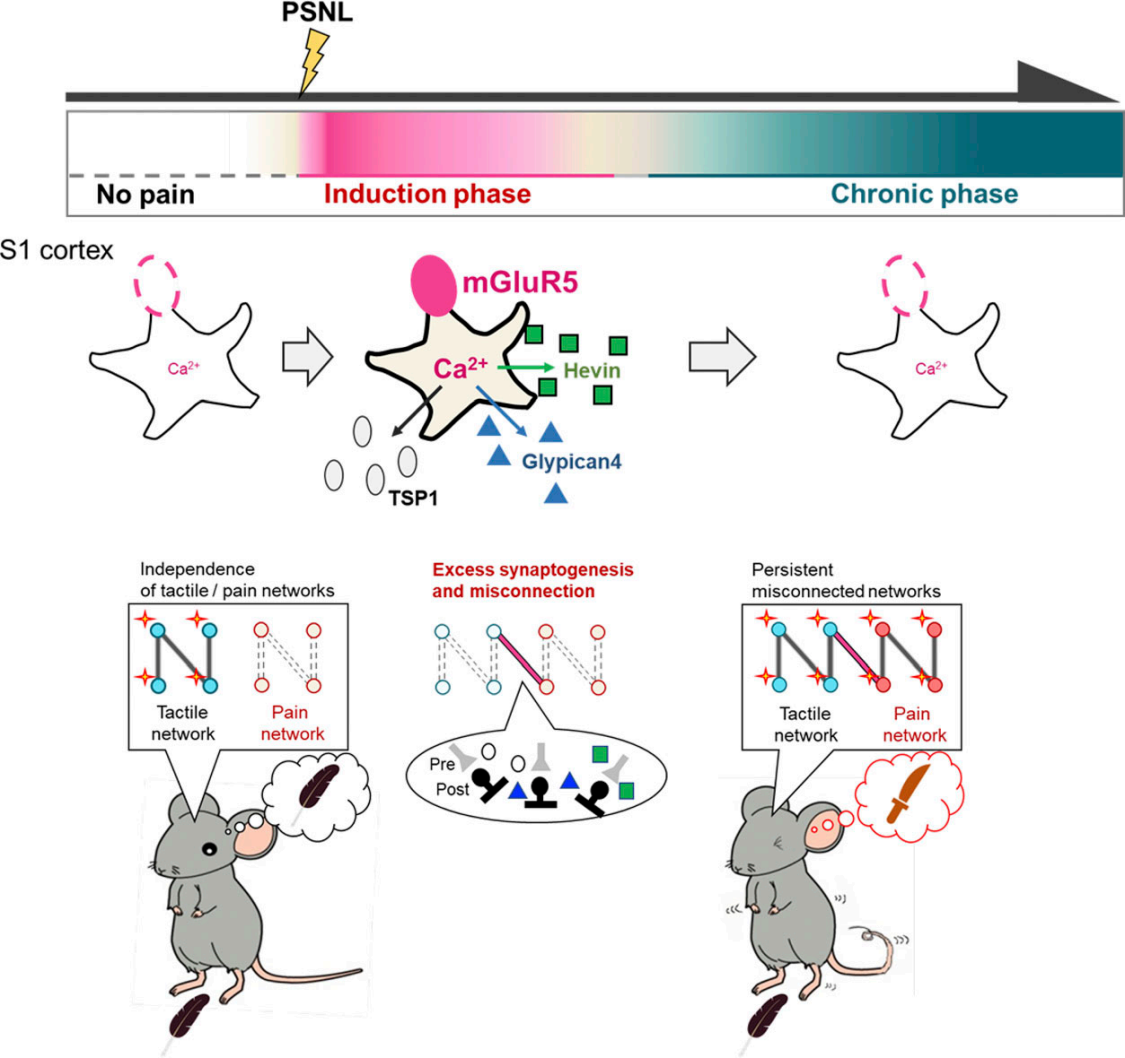
**Schematic of the reemergence of mGluR5 in S1 astrocytes, which causes a change in synapse remodeling.** Normal adult astrocytes do not express mGluR5. Soon after PSNL, mGluR5 transiently reemerges in S1 astrocytes during the induction phase of pain. The reemergence of S1 astrocytes induces (1) frequent Ca^2+^ signals in astrocytes, (2) the expression of multiple synaptogenic molecules, including TSP1, Glypican-4, and Hevin, (3) excess excitatory synaptogenesis and the inappropriate rewiring of S1 networks, and (4) misconnection of networks and neuropathic mechanical allodynia, which is persistent and lasts even after mGluR5 disappears.

Taken together, our findings suggest that the reemergence of mGluR5 in S1 astrocytes might function as a synaptic organizer of S1 cortical networks, leading to the misconnection of networks and mechanical allodynia (Fig. 8). In addition, the reemergence of astrocytic mGluR5 might control synaptic plasticity in the adult brain.

## Discussion

Using astro-mGluR5-cKO mice, we demonstrated that the transient reemergence of mGluR5 in S1 astrocytes was responsible for PSNL-induced synapse remodeling in the S1 cortex, leading to mechanical allodynia. mGluR5 increased Ca^2+^ signals in S1 astrocytes, followed by the expression of multiple synaptogenic molecules including TSP1, Glypican-4, and Hevin, which caused the formation of excessive excitatory synapses and long-lasting alteration of neuronal activity in the S1 cortex, ultimately leading to refractory neuropathic pain (Kim et al., 2016). A single molecule, mGluR5, was required for induction of the pain-forming cascades, even though its expression reemerged in the S1 cortex, a restricted brain region, in a specific cell type (astrocytes), and in a limited time frame during the induction phase of pain.

### Relevance of astro-mGluR5-cKO mice for the study of S1 astrocytes

The current study aimed to clarify the role of astrocytic mGluR5-mediated synaptic reorganization and rewiring of neuronal networks in the S1 cortex. We found that after PSNL, mGluR5 was transiently expressed in the hindlimb region of the S1 cortex and in the dorsal horn of spinal cord astrocytes (Fig. 1). On this basis, we generated astro-mGluR5-cKO mice by crossing GLAST-CreERT2 mice with floxed *Grm5* (encoding mGluR5) mice. GFAP-Cre mice are often used to drive astrocyte-specific Cre-recombination. However, they are not suitable for controlling cortical astrocyte gene expression, because the promoter activity is relatively low in the cortex (Fujita et al., 2014; Sloan and Barres, 2014; Su et al., 2004). In contrast, the GLAST promoter activity is relatively high in cortical astrocytes and is almost absent in spinal astrocytes (Borggrewe et al., 2021; Cahoy et al., 2008; Regan et al., 2007). Thus, crossing GLAST-CreERT2 mice with floxed-mGluR5 mice allowed us to delete genes in cortical astrocytes without affecting spinal cord astrocytes. PSNL actually enhanced mGluR5 expression in both contralateral S1 cortical astrocytes and ipsilateral spinal astrocytes in WT mice (Fig. 1 F). However, in astro-mGluR5-cKO mice, mGluR5 upregulation was abolished in S1 astrocytes but not in spinal astrocytes, and the expression level was similar to that in WT mice (Fig. 2 E). Therefore, using astro-mGluR5-cKO mice, we could determine whether and how brain astrocytic mGluR5 contributes to synaptic reorganization and induction of neuropathic pain.

### mGluR5 in S1 cortical astrocytes initiates pain cascades

The reemergence of astrocytic mGluR5 was transient, limited to 3–7 d, and disappeared 10 d after PSNL (Fig. 1). This time course was well associated with that of PSNL-evoked Ca^2+^ fluctuations in S1 astrocytes, TSP1 production, and excess synapse formation, which we reported previously (Kim et al., 2016; Kim et al., 2017). PSNL also increased extracellular glutamate levels in the S1 cortex, and glutamate acting on mGluR5 increased TSP1 in primary astrocyte culture via a Ca^2+^-dependent mechanism (Kim et al., 2016). Thus, we hypothesized that activation of mGluR5 in S1 astrocytes would trigger a series of pain cascades. Here, we produced astro-mGluR5-cKO mice and showed that all of the PSNL-evoked pain-related responses were abolished by deletion of mGluR5 in cortical astrocytes (Figs. 2, 3, 4, 5, 6, 7, and 8). Therefore, it is strongly suggested that mGluR5 in S1 astrocytes is a key factor that initiates S1 astrocyte-mediated synapse remodeling, network remodeling, and mechanical allodynia.

Regarding synapse formation, we previously used an in vivo spine imaging technique using Thy1-GFP mice. In the current study, we used immunohistochemical analysis by double staining of pre- and postsynaptic regions (Fig. 6). In vivo spine imaging can be used to monitor the temporal dynamics of morphological changes in the same spines by tracking them over time. In contrast, immunohistochemistry has the advantage of visualizing pre- and postsynaptic changes and analyzing the spatial changes involved in synaptic gain and loss in more detail. In this study, we confirmed that PSNL significantly increased the number of excitatory synapses in the S1 cortex in a limited time frame, i.e., the induction phase. Furthermore, differential staining of PSD95 for VGlut1 or VGlut2 indicated changes in synaptogenesis between intracortical and thalamocortical synapses (Kaneko and Fujiyama, 2002), respectively, and we found that both types of synapses (VGlut1/PSD95 and VGlut2/PSD95) were upregulated by PSNL (Fig. 6). Taken together, we concluded that the series of PSNL-evoked S1 astrocyte-mediated pain cascades was triggered by the reemergence of mGluR5. Although we cannot completely exclude the possibility that other mGluRs may take over the function of mGluR5, the findings that mGluR5 expression levels were associated with its functional changes, and that the mGluR5 antagonist inhibited astrocyte-mediated synapse remodeling and mechanical allodynia (Kim et al., 2016), strongly suggest that mGluR5 rather than other mGluRs would play a central role in these pain cascades.

### Activation of mGluR5 in S1 astrocytes triggers excessive synapse formation via expression of multiple synaptogenic molecules

Astrocytes express multiple synaptogenic molecules, including TSPs, Glypicans, and Hevin, which regulate the distribution of synapses during development and in the healthy brain and pathological conditions. Previously, we found that TSP1 in S1 astrocytes was upregulated in the induction phase of neuropathic pain (Kim et al., 2016). In addition to TSP1, the current study demonstrated that Glypican-4 and Hevin were also upregulated by PSNL, which was also dependent on astrocytic mGluR5. However, the proportions of TSP1-, Glypican-4–, and Hevin-expressing cells were completely different. PSNL increased the number of TSP1- or Glypican-4–expressing astrocytes (Fig. 4). In contrast, Hevin was expressed by almost all astrocytes in the nonpathogenic S1 cortex even though mGluR5 was absent (Fig. 4 F), and the expression level of each astrocyte was increased by PSNL. It is not clear whether single astrocytes increase expression of all synaptogenic molecules upon stimulation with mGluR5 or whether subpopulations of astrocytes differentially upregulate some synaptogenic molecules, producing astrocytic heterogeneity (Chai et al., 2017; John Lin et al., 2017).

The present finding that astrocytic mGluR5 evokes multiple synaptogenic molecules (TSP1, Glypican-4, and Hevin) suggests that these molecules may act cooperatively to promote excess synapse formation and rewiring of the S1 pain circuitry. However, mechanical allodynia and excess synaptogenesis were significantly inhibited by interfering with individual molecules via the administration of siRNA specific for TSP1 (Kim et al., 2016), Glypivan-4, or Hevin (Figs. 5 and 7). These findings may be explained as follows. (a) Each molecule may regulate a different step in synaptogenesis, and thus when even one molecule is absent, the overall response is reduced. (b) Each molecule may regulate common steps in synaptogenesis in parallel, and thus when one molecule is absent, all responses are decreased because of a lack of an additive effect. We do not yet have answers to these questions, but it is likely that both (a) and (b) are relevant, because each molecule has its own unique and common functions. For example, TSP1 induces excitatory synapses by binding to multiple postsynaptic receptors including α2δ-1 (Eroglu et al., 2009), ApoER2 (Blake et al., 2008), or neuroligin1 (Xu et al., 2010). Hevin also binds to neuroligins including neuroligin1 (Singh et al., 2016). Hevin additionally bridges postsynaptic neuroligins and presynaptic neurexin1α, which promotes *N*-methyl-D-aspartate receptor recruitment to postsynaptic regions in vivo (Singh et al., 2016). Synapses formed by TSP1 or by Hevin alone are ultrastructurally normal and presynaptically active but postsynaptically silent in vitro. Glypican-4, however, was identified as a functional synaptogenic molecule released by astrocytes that strengthened glutamatergic synapses by recruiting GluA1-containing α-amino-2,3-dihydro-5-methyl-3-oxo-4-isoxazolepropanoic acid receptors (Allen et al., 2012). Glypican-4 interacts with presynaptic type 2a receptor protein tyrosine phosphatase δ, which induces the release of neuronal pentraxin1 from neurons (Farhy-Tselnicker et al., 2017). How astrocytic mGluR5 uses these molecules differently to regulate synaptic remodeling remains a major question for future study.

Recently, it was reported that astrocytes in the striatum mediated hyperactivity and disturbances of attention by increasing corticostriatal synaptogenesis (Nagai et al., 2019). For these actions, only astrocytic TSP1, but not Glypican-4 or Hevin, was involved, i.e., medium spiny neurons release γ-amino butyric acid (GABA) to activate GABA_B_ receptor–mediated Ca^2+^ signals in striatal astrocytes, leading to TSP1 production and synaptogenesis. This discrepancy may be explained by differences in (a) the receptors that trigger the series of responses, i.e., Gi-coupled GABA_B_ receptors and Gq-coupled mGluR5; (b) the brain regions, i.e., the striatum and S1 cortex; and (c) the physiological and pathological conditions. Regarding (c), the GABA_B_ receptor is present in healthy striatal astrocytes, whereas mGluR5 is absent under physiological conditions in adults, and it reemerges in S1 cortical astrocytes only under pathological conditions. Therefore, astrocytic mGluR5 and subsequent Ca^2+^ responses might be a specialized critical cue that facilitates stronger synaptogenesis in the adult brain compared with physiological conditions (Anderson et al., 2016; Burda et al., 2016). However, further studies are required for clarification.

### Reemergence of astrocytic mGluR5 in the S1 cortex controls the critical time frame for the pathogenesis of neuropathic pain

As mentioned above, enhanced Ca^2+^ signaling in S1 astrocytes, the production of synaptogenic molecules, and synaptic remodeling were mGluR5 dependent, and all of these events were restricted to the induction phase of pain. However, mechanical allodynia persisted after this phase (Fig. 1), suggesting that the neuronal networks established by this transient astrocyte-dependent synaptic and network reorganization remain stable thereafter. Supporting our hypothesis, the proportion of c-fos–expressing neurons in the S1 cortex, especially in layers II/III, was increased, even at 1 month after PSNL, and was dependent on astrocytic mGluR5 (Fig. 6). This suggests that the stability of the established circuit might be the cause of the intractability of neuropathic pain. This finding also indicates that synaptic plasticity might be flexibly controlled, even in adult brains, if these astrocytic functions can be controlled precisely and appropriately. Because the reemergence of mGluR5 in adult astrocytes is often associated with various pathological conditions (Buscemi et al., 2017; Grolla et al., 2013; Iyer et al., 2014; Shrivastava et al., 2013; Umpierre et al., 2016), including mechanical allodynia, as demonstrated in this study, astrocytic mGluR5 is considered to be a pathogenic molecule in the adult brain. However, in the early postnatal development stage, astrocytes strongly express mGluR5 (Cahoy et al., 2008; Rusnakova et al., 2013; Sun et al., 2013; Tanaka et al., 2021) and various synaptogenic molecules including TSP1, Glypican-4, and Hevin (Farhy-Tselnicker et al., 2017), which are thought to be related to synaptic plasticity during the so-called critical period. Therefore, if the synaptic environment can be appropriately controlled, mGluR5-mediated synaptogenesis might be a beneficial function because synaptic plasticity can be dramatically increased, even in the adult brain. This study focused on the synaptogenic roles of astrocytic mGluR5 in the adult pathological brain, but these pathways might also be closely related to those in the healthy postnatal developing brain, and therefore, mGluR5 may also control a “critical period” of postnatal development.

In conclusion, using well-developed astro-mGluR5-cKO mice, we demonstrated that the reemergence of mGluR5 in S1 astrocytes is a key factor that initiates a series of astrocyte-mediated pain cascades induced by PSNL. The reemergence of mGluR5 after PSNL had a very limited spatiotemporal pattern, i.e., in astrocytes in the S1 cortex and in the induction phase of pain. However, this limited expression of mGluR5 produced multiple synaptogenic molecules, leading to a marked increase in synaptic plasticity, even in the adult brain, which could be related to the etiology of intractable mechanical allodynia caused by neural network reorganization.

## Materials and methods

### Animals and drug administration

All procedures were performed in accordance with the guidelines and previous approval of the Animal Care Committee of the University of Yamanashi. WT mice (C57Bl/6) were purchased from Japan SLC (Hamamatsu). GLAST-CreERT2 mice were kindly provided by Dr. Magdalena Götz (Helmholz Center Munich, Institute of Stem Cell Research, Munich, Germany) and by Dr. K. Tanaka (Tokyo Medical and Dental University, Tokyo, Japan), and mGluR5^flx/flx^ mice were kindly provided by Dr. Anis Contractor (Northwestern University, Evanston, IL). We crossed GLAST-CreERT2 mice with mGluR5^flx/flx^ mice to obtain astro-mGluR5-cKO mice. GLAST-CreERT2^−/−^::mGluR5^flx/flx^ mice were used as controls. GLAST-CreERT2^−/+^::GCaMP3^flx-STOP-flx^ mice were used for Ca^2+^ imaging by two-photon microscopy. In these experiments, GLAST-CreERT2^−/+^::GCaMP3^flx-STOP-flx^::mGluR5^WT/WT^ mice were used as a control group, and GLAST-CreERT2^−/+^::GCaMP3^flx-STOP-flx^::mGluR5^flx/flx^ mice were used for the cKO group. Transgenic mice were i.p. injected with tamoxifen (100 mg/kg, dissolved in corn oil). A schematic timeline for the drug administration is shown in Fig. 3 A. All mice were back-crossed to C57BL/6 mice.

### Surgical procedure and behavioral assessment

Surgery was performed on 2–3-mo-old male mice under isoflurane anesthesia. One third to one half of the right sciatic nerve was ligated with an 8-0 suture. In sham-operated mice, the nerve was exposed without ligation. On each testing day, mice were habituated for 45 min in a plastic box covered with red transparent vinyl with a wire mesh floor before testing. Mechanical allodynia was quantified by applying a 0.16-g von Frey filament to the plantar surface and scoring the hind paw withdrawal response to the filament as previously described (Chaplan et al., 1994). Briefly, the hind paw response score was evaluated as follows: 0, no response; 1, withdrawal response away from the stimulus with slight flinching; and 2, intense withdrawal response away from the stimulus with brisk flinching and/or licking. One trial involved 10 applications of filaments every 2–5 min, each of which was scored as 0, 1, or 2. The trial was evaluated on the basis of a total score of 0–20 at the culmination of the test (percentage of maximum score). The experiments were performed under blind conditions.

### Immunohistochemistry

Mice were transcardially perfused with saline under anesthesia (induced by pentobarbital, 70 mg/kg, i.p.). The brain was dissected and immersion-fixed in 4% paraformaldehyde overnight at 4°C. After cryoprotection in 20% sucrose, the brain was embedded in optimum cutting temperature compound (Sakura Finetek) and stored at −80°C. Coronal sections (20 µm thick) were prepared using a cryostat (Leica), and free-floating sections were washed two times with PBS. These were then incubated at 4°C with blocking buffer consisting of 0.1% gelatin and 0.2% Triton X-100 in PBS for 45 min, followed by incubation with primary antibodies: anti-GFAP (1:1,000; Thermo Fisher Scientific), anti-GLAST (1:1,000; Miltenyi Biotec), anti-GFP (1:1,000; Thermo Fisher Scientific), anti-Iba1 (1:1,000; Wako), anti-NeuN (1:1,000; Millipore), anti-NG2 (1:1,000; Millipore), anti-S100β (1:1,000; Sigma-Aldrich), anti-Aldh1l1 (1:500; Millipore), anti–Glypican-4 (1:250; Proteintech), anti-Hevin (1:1,000; R&D Systems), anti-VGluT1 (1:2,500; Synaptic Systems), anti-VGluT2 (1:2,500; Millipore), or anti-PSD95 (1:500; Thermo Fisher Scientific), diluted in PBS. After washing twice with PBS, the sections were incubated with secondary antibodies (1:1,000; Invitrogen) for 1 h at room temperature. Slices were washed two times and mounted onto glass slides using mounting medium (Vectashield; Vector Laboratories). Confocal images were acquired on a FV1200 microscope (Olympus). Heat-induced antigen retrieval was performed for Glypican-4 and Aldh1l1 for 20 min at 95°C.

In Figs. 6 and 7, it was not possible to acquire images of all regions at once because of the large area of the brain observed in both cases. Therefore, we divided the regions and acquired images of each region, and later combined them by tiling. Therefore, the images are not uniform and look like splice images. When using a low-magnification objective lens, the spatial resolution is not sufficient and the analysis of the c-fos signal is insufficient.

### Immunohistochemical staining for mGluR5 with GFAP

Mice were transcardially perfused with saline under anesthesia (induced by pentobarbital, 70 mg/kg, i.p.). Brains were frozen in isopentane cooled with dry ice (−20°C), and coronal sections (20 µm thick) prepared using a cryostat (Leica) were placed on microscope slides, stored, and dried at −80°C in the presence of a desiccant. The slices were washed twice with PBS and incubated with blocking buffer consisting of 0.1% gelatin and 0.2% Triton X-100 in PBS for 45 min. The sections were incubated overnight at 4°C with primary antibodies against GFAP (1:1,000; Thermo Fisher Scientific) and mGluR5 (1:1,000; Millipore) diluted in PBS. After washing twice with PBS, the sections were incubated with secondary antibodies (1:1,000; Invitrogen) for 1 h at room temperature. Slices were washed twice and mounted onto glass slides using mounting medium (Vectashield; Vector Laboratories).

### Immuno-EM

Ultrastructural investigation of mGluR5 expression in astrocytes was carried out by pre-embedding immunogold EM as described previously (Parajuli et al., 2012), with some modifications. Deeply anesthetized mice were transcardially perfused with 25 mM PBS for 1 min followed by a fixative containing 4% paraformaldehyde, 0.05% glutaraldehyde, and 15% saturated picric acid in 0.1 M phosphate buffer for 12 min (total 50 ml). Coronal sections of the S1 cortical region were cut with a microslicer (Linear 7 Pro; Dosaka) at a thickness of 60 µm. These sections were cryoprotected with 30% sucrose in phosphate buffer, freeze-thawed several times with liquid nitrogen, blocked for 1 h in Tris-buffered saline containing 10% normal goat serum, and incubated with an anti-mGluR5 guinea pig antibody (2 µg/ml; Frontier Institute) in Tris-buffered saline containing 1% normal goat serum for 48 h at 4°C. After washing, the sections were incubated with a 1.4-nm gold particle–coupled anti-guinea pig secondary antibody (Nanoprobes) diluted in Tris-buffered saline at a ratio of 1:100 overnight at 4°C. After washing, the sections were postfixed in 1% glutaraldehyde for 10 min, followed by gold enhancement of the immunogold particles using a gold enhance-EM kit (Nanoprobes). The sections were postfixed with 0.5% osmium tetroxide for 40 min, en bloc counterstained with 1% uranyl acetate for 40 min, and dehydrated in a graded ethanol series followed by propylene oxide treatment. The sections were infiltrated overnight at room temperature in Durcupan resin (Sigma-Aldrich) and transferred to glass slides for flat embedding. After resin curing at 60°C, the trimmed tissues from the region of interest (layers I to II/III of S1 in the left hemisphere) were reembedded in Durcupan resin blocks for ultrathin sectioning. The ultrathin sections (60-nm thickness) were cut within 5 µm of the section surface and collected on pioloform-coated single-slot copper grids. The analyzed micrographs were obtained at 4,000× by a charge-coupled device camera that was connected to a transmission electron microscope (H-7650; Hitachi High-Technologies).

### Two-photon Ca^2+^ imaging

Acute coronal cortical slices including the S1HL region (300 µm) were cut in a solution consisting of (in mM): 92 NaCl, 2.5 KCl, 1.2 NaH_2_PO_4_, 30 NaHCO_3_, 20 Hepes, 25 glucose, 5 C_6_H_7_NaO_6_, 2 thiourea, 3 sodium pyruvate, 10 MgSO_4_, and 0.5 CaCl_2_, saturated with 95% O_2_ and 5% CO_2_. Slices were incubated at 34°C for 10 min in a solution consisting of (in mM): 93 N-Methyl-D(-)-glucamine, 93 HCl, 2.5 KCl, 1.2 NaH_2_PO_4_, 30 NaHCO_3_, 20 Hepes, 25 glucose, 5 sodium ascorbate, 2 thiourea, 3 sodium pyruvate, 10 MgSO_4_, and 0.5 CaCl_2_, saturated with 95% O_2_ and 5% CO_2_. Slices were stored at room temperature in artificial cerebrospinal fluid consisting of (in mM): 124 NaCl, 2.5 KCl, 1.2 NaH_2_PO_4_, 24 NaHCO_3_, 5 Hepes, 12.5 glucose, 2 MgSO_4_, and 2 CaCl_2_, saturated with 95% O_2_ and 5% CO_2_. Images were acquired at 1 frame/s for 201 s with a two-photon microscope (FV1000MPE; Olympus) using a Mai Tai laser (Spectra Physics) at 920 nm, through a 40× water-immersion objective lens. Astrocytes were selected from layer I in the S1HL and were typically 50 µm from the slice surface. Imaging data were analyzed using ImageJ (National Institutes of Health) and Origin software (OriginLab). Regions of interest were placed on astrocytic soma revealed by maximum-intensity projection of the stacked image. Within a region of interest, the GCaMP3 intensity was analyzed as Δ*F*/*F*_0_. Spontaneous Ca^2+^ fluctuations were detected semiautomatically using Origin and then manually verified.

### Quantification of synapses by immunohistochemistry

To quantify the excitatory synapse number in the S1 cortex, we followed the method previously described by Ippolito and Eroglu (2010). Three coronal sections per animal were stained with pre- and postsynaptic markers as described above, and 5-µm-thick confocal z-stacks (interval, 0.33 µm; 15 sections/z-stack; imaged area/scan, 2,726.93 µm^2^; 1,024 × 1,024 pixels) were taken at layer I of the ipsilateral and contralateral S1HL cortex using a 60× magnification objective lens (4.0× zoom). Maximum projections of three consecutive optical sections were generated from the original z-stack, and analyses were performed using the Puncta Analyzer plugin.

### Western blotting

To prepare S1HL samples, mice were transcardially perfused with saline under anesthesia. The S1HL cortex was dissected rapidly, immersed in Laemmli buffer, and lysed using a sonicator. Proteins were separated on 10–15% polyacrylamide gels and transferred to membranes. The membranes were incubated with blocking solution containing Blockace (0.4 mg/ml; DS Pharmabiomedical) and rinsed with Tris-buffered saline containing 0.1% Tween 20 (TBST). The membranes were incubated with primary antibodies, including Hevin (1:2,000; R&D Systems) or Glypican-4 (1:250; Proteintech), and diluted in TBST overnight or with β-actin (1:20,000; Sigma-Aldrich) for 8 h at 4°C. After washing with TBST twice, the membranes were incubated with secondary antibodies (HRP-conjugated anti-goat, anti-rabbit, or anti-mouse IgG; 1:10,000) for 1 h at room temperature. The membranes were washed with TBST twice, and detection was performed using a Super Signal West Femto kit (Thermo Fisher Scientific). Images were acquired using an LAS-4000 (Fujifilm).

### In vivo siRNA injection into the S1HL cortex

Mice were deeply sedated with isoflurane and stereotactically injected with 1.2 μl of an 8-µM siRNA solution using JetSi (Polyplus Transfection) within the left S1 cortex hindlimb region (0.5 mm posterior to the bregma, 1.5 mm lateral to the midline, 0.15 mm from the pial surface). siRNA solutions were prepared according to the manufacturer’s protocols. All siRNAs were purchased from Santa Cruz Biotechnology (control siRNA, sc 37007; *Thbs1*, sc-3666; *Gpc4* siRNA, sc-145457; *Sparcl1* siRNA, sc-153239). siRNA injection experiments were performed immediately before the sciatic nerve ligation surgery.

### Statistics

Data are presented as means ± SEM. Detailed statistical analysis methods are shown in the corresponding figure legends. All graphs and statistical tests were performed using Origin software (OriginLab). P values <0.05 were considered statistically significant.

### Online supplemental material

Fig. S1 shows that mGluR5 emergence is specific to S1 astrocytes by electron micrographs and immunohistochemistry. Fig. S2 shows that functions of astro-mGluR5-cKO astrocytes are normal by Ca^2+^ imaging. Fig. S3 shows that expression of Glypican-4 and Hevin is transient.

### Data and materials availability

The data that support the findings of this study are available from the corresponding author upon reasonable request.

## Supplementary Material

SourceData F4 contains original blots for Fig. 4.

SourceData F5 contains original blots for Fig. 5.

SourceData FS3 contains original blots for Fig. S3.
