# Supplementary figures and images for: Transient astrocytic mGluR5 expression drives synaptic plasticity and subsequent chronic pain in mice
Source: J Exp Med. 2022 Mar 23;219(4):e20210989. doi: 10.1084/jem.20210989 (PMC8952801; doi:10.1084/jem.20210989)

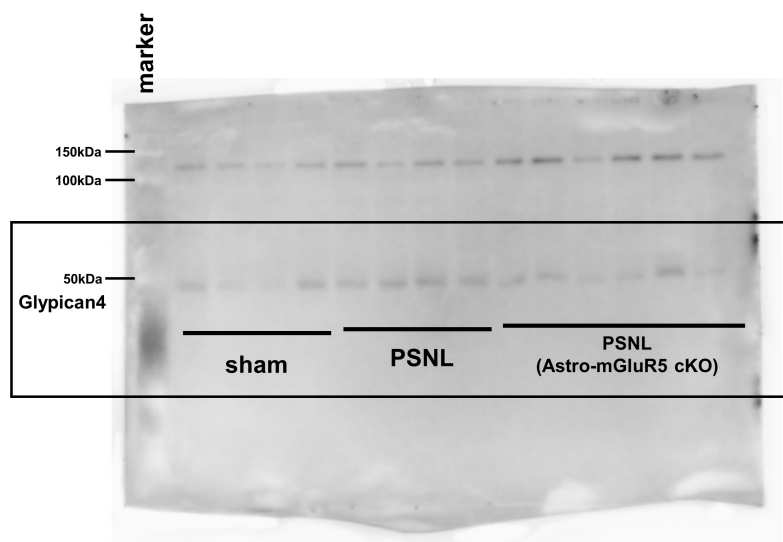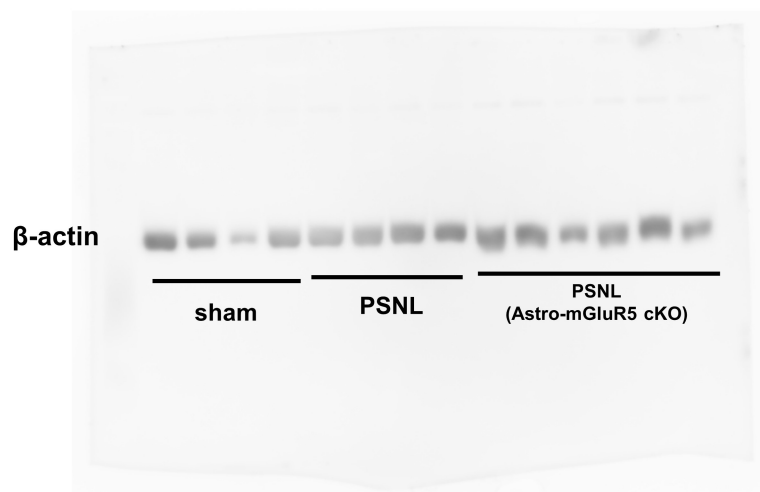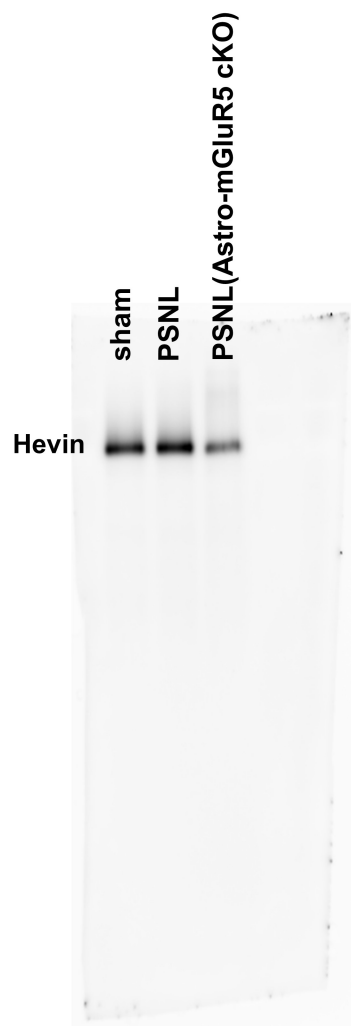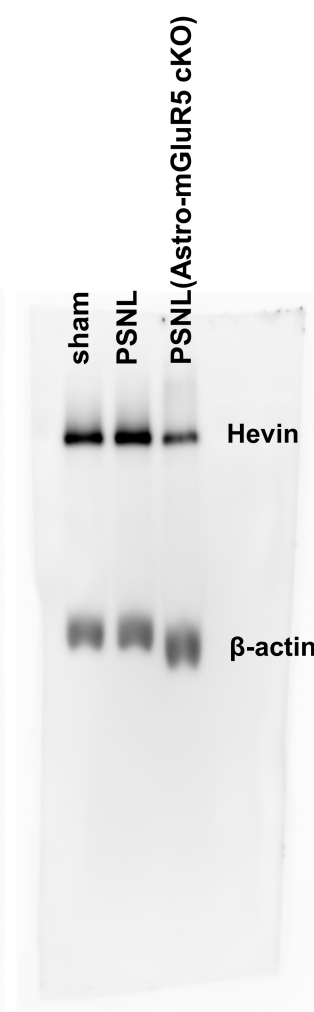

Related to Fig. 4 Danjo, et al.

Supplement: SourceData F4 — contains original blots for Fig. 4. [file JEM_20210989_SourceDataF4.pdf]

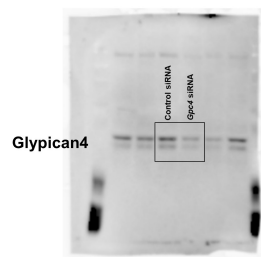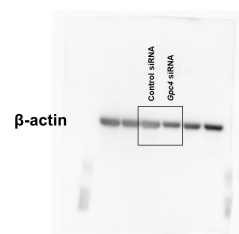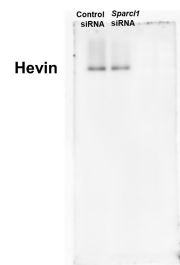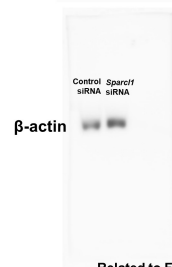

Related to Fig. 5 Danjo, et al.

Supplement: SourceData F5 — contains original blots for Fig. 5. [file JEM_20210989_SourceDataF5.pdf]

Glypican4

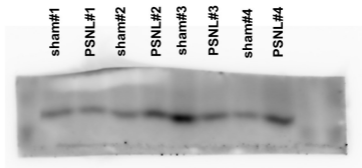

Hevin

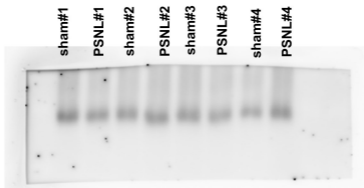

$\beta$ -actin

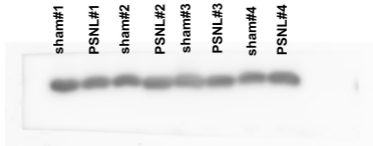

Supplement: SourceData FS3 — contains original blots for Fig. S3. [file JEM_20210989_SourceDataFS3.pdf]
